# T helper 2 cell–directed immunotherapy eliminates precancerous skin lesions

**DOI:** 10.1172/JCI183274

**Published:** 2025-01-02

**Authors:** Tomonori Oka, Sabrina S. Smith, Heehwa G. Son, Truelian Lee, Valeria S. Oliver-Garcia, Mahsa Mortaja, Kathryn E. Trerice, Lily S. Isakoff, Danielle N. Conrad, Marjan Azin, Neel S. Raval, Mary Tabacchi, Luni Emdad, Swadesh K. Das, Paul B. Fisher, Lynn A. Cornelius, Shadmehr Demehri

**Affiliations:** 1 Center for Cancer Immunology and Cutaneous Biology Research Center, Krantz Family Center for Cancer Research and Department of Dermatology, Massachusetts General Hospital, Boston, Massachusetts, USA.; 2 Division of Dermatology, Department of Medicine, Washington University School of Medicine, St. Louis, Missouri, USA.; 3 Department of Human and Molecular Genetics, Virginia Commonwealth University, School of Medicine, Richmond, Virginia, USA.; 4 VCU Institute of Molecular Medicine, Virginia Commonwealth University, School of Medicine, Richmond, Virginia, USA.; 5 VCU Massey Comprehensive Cancer Center, Virginia Commonwealth University, School of Medicine, Richmond, Virginia, USA.; 6 Department of Dermatology, Massachusetts General Hospital and Harvard Medical School, Boston, Massachusetts, USA.

**Keywords:** Dermatology, Oncology, Immunotherapy, Skin cancer, T cells

## Abstract

The continuous rise in skin cancer incidence highlights an imperative for improved skin cancer prevention. Topical calcipotriol-plus–5-fluorouracil (calcipotriol-plus–5-FU) immunotherapy effectively eliminates precancerous skin lesions and prevents squamous cell carcinoma (SCC) in patients. However, its mechanism of action remains unclear. Herein, we demonstrate that calcipotriol-plus–5-FU immunotherapy induces T helper type 2 (Th2) immunity, eliminating premalignant keratinocytes in humans. CD4^+^ Th2 cells were required and were sufficient downstream of thymic stromal lymphopoietin cytokine induction by calcipotriol to suppress skin cancer development. Th2-associated cytokines induced IL-24 expression in cancer cells, resulting in toxic autophagy and anoikis followed by apoptosis. Calcipotriol-plus–5-FU immunotherapy was dependent on IL-24 to suppress skin carcinogenesis in vivo. Collectively, our findings establish a critical role for Th2 immunity in cancer immunoprevention and highlight the Th2/IL-24 axis as an innovative target for skin cancer prevention and therapy.

## Abstract 2

T helper 2 cells that are activated by calcipotriol plus 5-FU immunotherapy eliminate premalignant skin lesions in humans.

## Graphical abstract

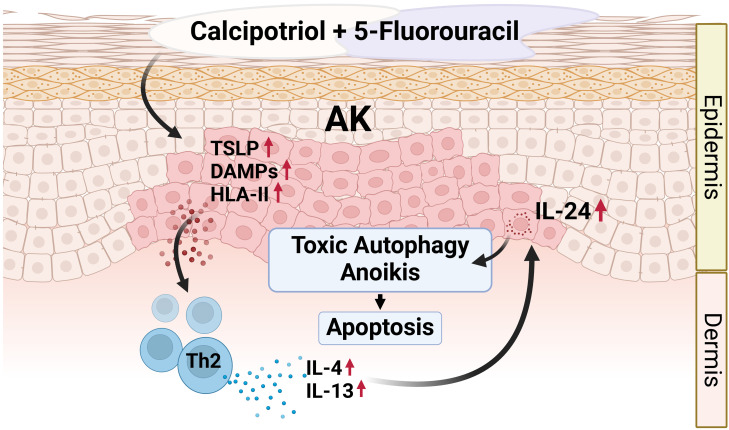

## Introduction

Despite significant advances in cancer therapeutics led by innovations in immunotherapy, current cancer therapeutics have prohibitive side effects and costs for use in cancer prevention. Hence, effective strategies for cancer immunoprevention are urgently needed. Cutaneous squamous cell carcinoma (SCC) is the second most common cancer, which can cause substantial morbidity, mortality, and economic burden (1). SCC is a highly immunogenic cancer that is immune-regulated from its inception (2). Importantly, actinic keratosis (AK), a precursor to SCC, can be identified and treated to prevent SCC. Patients with multiple AKs have a relatively high cumulative skin cancer risk (3). Current AK field treatments include topical 5-fluorouracil (5-FU), photodynamic therapy, imiquimod, and tirbanibulin (4–7). Although they can eliminate AKs, only 5-FU has been proven to reduce the risk of SCC within 1 year after treatment, and this benefit is no longer apparent 2 years after treatment (8). The impact of other AK field treatments on SCC prevention is unknown. By contrast, AK immunotherapy with the aim of SCC immunoprevention provides an innovative, attainable strategy.

We have previously demonstrated the high efficacy of topical calcipotriol-plus–5-FU therapy for eliminating AKs through a randomized, double-blind clinical trial (9). Calcipotriol is an FDA-approved low-calcemic vitamin D analog used for psoriasis treatment (10). Calcipotriol induces the expression of thymic stromal lymphopoietin (TSLP) in keratinocytes (9, 11, 12). 5-FU synergizes with calcipotriol to generate an immune-mediated modality for AK clearance. Calcipotriol-plus–5-FU treatment promotes the induction of TSLP in AK keratinocytes, leading to massive T cell infiltration and tissue-resident memory T (T_RM_) cell formation in AKs (9, 13). Importantly, topical calcipotriol-plus–5-FU treatment has shown efficacy in preventing SCC within 3 years after treatment (13). Because calcipotriol-plus–5-FU treatment activates the adaptive immune system to eliminate AKs and helps prevent SCC (9, 13), this topical immunotherapy highlights an innovative therapeutic strategy for AK treatment distinct from current cytotoxic treatments for AK. However, the precise nature of the antitumor immunity induced by calcipotriol-plus–5-FU treatment and its effector mechanism(s) in humans remain unknown.

Herein, we investigated the mechanism of calcipotriol-plus–5-FU immunotherapy in eliminating premalignant keratinocytes. Through an open-label clinical trial, we found that CD4^+^ T helper 2 (Th2) cells were the dominant immune cells infiltrating AKs after calcipotriol-plus–5-FU treatment. Th2-polarized CD4^+^ T cells responding to TSLP played a seminal role in tumor suppression in vivo. Calcipotriol-plus–5-FU immunotherapy induced cell death in AKs via toxic autophagy and anoikis followed by apoptosis. IL-24 (melanoma differentiation–associated gene-7 or MDA-7) was a downstream effector molecule induced by Th2-associated cytokines, IL-4 and IL-13 (14–16), that promoted toxic autophagy and apoptosis in cancer cells (14, 15, 17). IL-24 was essential for calcipotriol-plus–5-FU immunotherapy to mediate skin cancer protection in vivo. Our findings highlight Th2 immunity as a promising target for skin cancer prevention and treatment.

## Results

### Calcipotriol-plus–5-FU immunotherapy trial.

**Figure 1:**
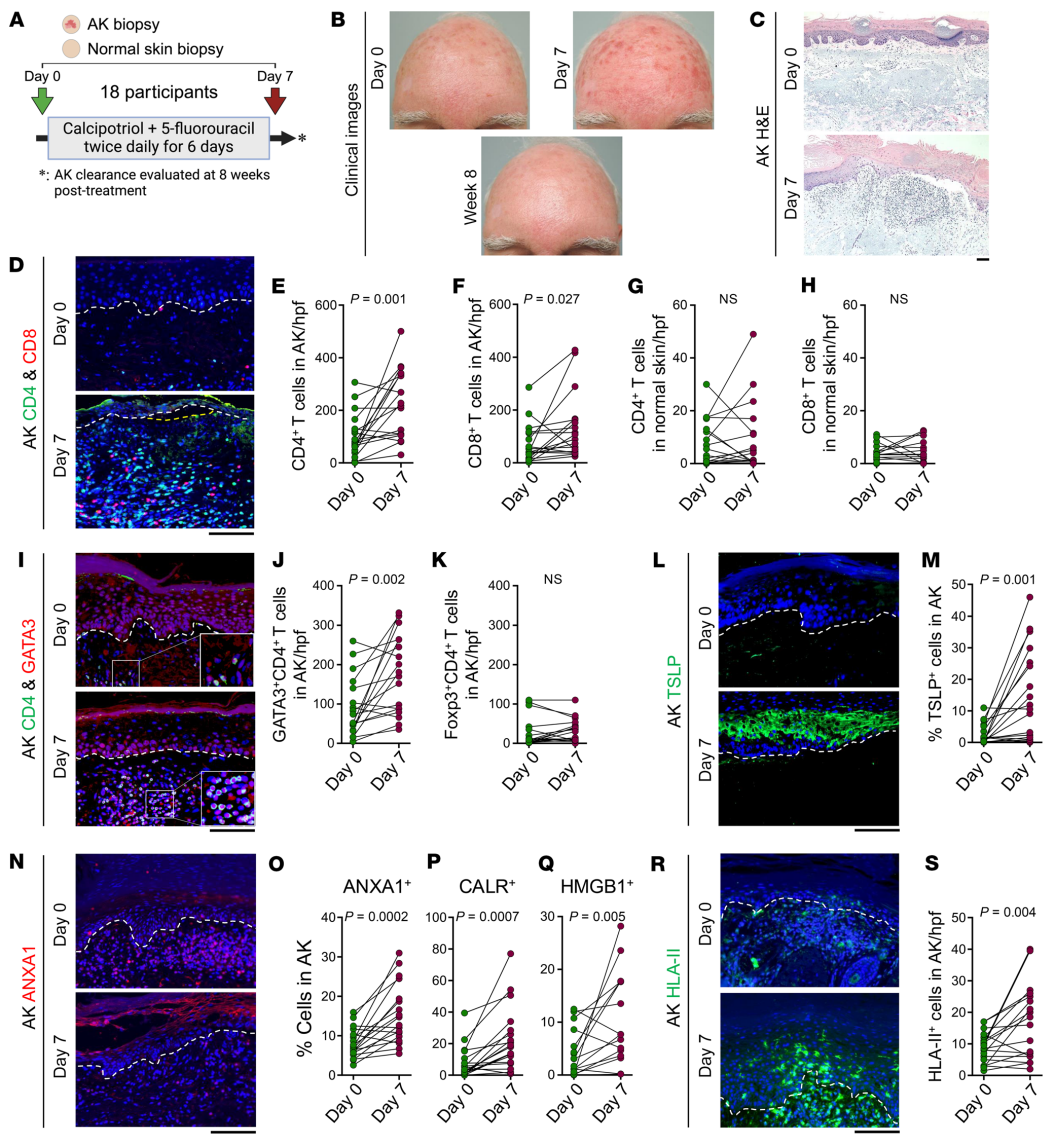
Calcipotriol-plus–5-FU immunotherapy induces robust Th2 immunity in AKs associated with TSLP and DAMP upregulation in keratinocytes. (**A**) Schematic diagram of calcipotriol-plus–5-FU immunotherapy open-label trial. (**B**) Representative clinical photographs of skin treated with calcipotriol-plus–5-FU. Photographs were taken before (day 0), and after treatment (day 7 and week 8). (**C**) Representative H&E-stained AKs before (day 0) and after (day 7) calcipotriol-plus–5-FU treatment. (**D**) Representative images of CD4/CD8-stained AKs before (day 0) and after (day 7) calcipotriol-plus–5-FU treatment. Note that CD4^+^ and CD8^+^ cells are CD3^+^ T cells. (**E**–**H**) Quantification of CD4^+^ T cells in AKs (**E**), CD8^+^ T cells in AKs (**F**), CD4^+^ T cells in normal skin (**G**), and CD8^+^ T cells in normal skin (**H**) before (day 0) and after (day 7) calcipotriol-plus–5-FU treatment. (**I**) Representative images of CD4/GATA3-stained AKs before (day 0) and after (day 7) calcipotriol-plus–5-FU treatment. Note that GATA3^+^CD4^+^ cells are CD3^+^ T cells. (**J** and **K**) Quantification of GATA3^+^CD4^+^ T cells (**J**) and Foxp3^+^CD4^+^ T cells (**K**) in AKs before (day 0) and after (day 7) calcipotriol-plus–5-FU treatment. (**L**) Representative images of TSLP-stained AKs before (day 0) and after (day 7) calcipotriol-plus–5-FU treatment. (**M**) Quantification of TSLP^+^ cells as percentage DAPI^+^ keratinocytes in AKs before (day 0) and after (day 7) calcipotriol-plus–5-FU treatment. (**N**) Representative images of ANXA1-stained AKs before (day 0) and after (day 7) calcipotriol-plus–5-FU treatment. (**O**–**Q**) Quantification of ANXA1^+^ cells (**O**), CALR^+^ cells (**P**), and HMGB1^+^ cells (**Q**) as percent DAPI^+^ keratinocytes in AKs before (day 0) and after (day 7) calcipotriol-plus–5-FU treatment. (**R**) Representative images of HLA-II–stained AKs before (day 0) and after (day 7) calcipotriol-plus–5-FU treatment. (**S**) Quantification of HLA-II^+^ cells as percentage DAPI^+^ cells in AKs. Each dot represents an AK or normal skin sample. *n* = 18 participants at each time point; paired *t* test. Dashed lines mark the epidermal basement membrane in immunofluorescence images. Scale bars: 100 μm.

We performed an open-label clinical trial to investigate the mechanism of calcipotriol-plus–5-FU immunotherapy. Eighteen patients with AKs who met the eligibility criteria were enrolled in the study (Supplemental Figure 1A and Supplemental Table 1; supplemental material available online with this article; https://doi.org/10.1172/JCI183274DS1). All the participants applied 0.0025% calcipotriol-plus–2.5% 5-FU field treatment to the entirety of their qualified anatomical sites, face, scalp, right upper extremity (RUE) and/or left upper extremity (LUE), twice daily for 6 days (Supplemental Figure 1B). All the participants completed the treatment course and underwent clinical evaluation and AK/normal skin biopsies before treatment (day 0), 1 day after the last treatment (day 7), and 8 weeks after treatment (Figure 1A and Supplemental Figure 1, A and B). Topical calcipotriol-plus–5-FU immunotherapy led to a mean reduction in the number of AKs of 95% on the face, with 7 out of 10 participants showing complete clearance, 82% on the scalp, 65% on the RUE, and 68% on the LUE 8 weeks after treatment (Supplemental Figure 1, C and D). Calcipotriol-plus–5-FU immunotherapy induced profound erythema centered around the AKs after treatment, followed by a resolution of skin erythema and elimination of AKs by week 8 (Figure 1B). All skin reactions were resolved by week 4 after treatment.

### Calcipotriol-plus–5-FU immunotherapy induces robust Th2 immunity in AKs.

Calcipotriol-plus–5-FU treatment induced massive immune cell infiltration in AKs, dominated by CD4^+^ T and, to a lesser extent, CD8^+^ T cells (Figure 1, C–F). By contrast, calcipotriol-plus–5-FU immunotherapy did not cause T cell infiltration into the normal skin (Figure 1, G and H, and Supplemental Figure 2, A and B). GATA3^+^CD4^+^ Th2 cells were markedly increased in AKs after treatment (Figure 1, I and J). Foxp3^+^CD4^+^ regulatory T cell number was not changed in AKs after treatment (Figure 1K and Supplemental Figure 2C). Calcipotriol-plus–5-FU immunotherapy did not affect GATA3^+^CD4^+^ Th2 or Foxp3^+^CD4^+^ regulatory T cell numbers in the normal skin (Supplemental Figure 2, D and E). The other CD4^+^ T cell subsets, T-bet^+^CD4^+^ Th1 and RORγt^+^CD4^+^ Th17 cells, were rare in AKs before and after treatment (Supplemental Figure 2, F and G). Calcipotriol-plus–5-FU immunotherapy also induced CD103^+^CD4^+^ resident memory T cell formation in AKs (Supplemental Figure 2H) (18). IHC staining further confirmed that GATA3^+^CD4^+^ Th2 cells were the dominant cell type in AKs treated with calcipotriol-plus–5-FU while cytotoxic molecules, Perforin and Granzyme B, SLAMF7, a cytotoxicity-related transcription factor, and T-bet were rarely detected (Supplemental Figure 2I). These results indicate that Th2 cells are the primary effector T cells activated by calcipotriol-plus–5-FU immunotherapy in AKs.

### Calcipotriol-plus–5-FU immunotherapy upregulates TSLP and damage-associated molecular patterns in the premalignant keratinocytes.

To determine the upstream activators of Th2 immunity in AKs, we investigated the immune factors induced by calcipotriol-plus–5-FU treatment in AK keratinocytes. Calcipotriol-plus–5-FU immunotherapy upregulated TSLP in premalignant keratinocytes but did not affect TSLP expression in the normal skin (Figure 1, L and M, and Supplemental Figure 3, A and B) (19). Interestingly, TSLP was detectable in the plasma of 3 out of 4 participants who treated their face, scalp, RUE, and LUE with calcipotriol-plus–5-FU (Supplemental Figure 3C). As calcipotriol induced TSLP expression in AKs but not in the normal human skin, we investigated whether calcipotriol specifically induced TSLP in malignant keratinocytes. Calcipotriol induced *TSLP* expression only in SCC cells but not in the normal keratinocyte cell lines (Supplemental Figure 3, D–G). Damage-associated molecular patterns (DAMPs) released in response to cellular stress and death are potent immune activators (20). Among them, Annexin A1 (ANXA1), calreticulin (CALR), and High Mobility Group Box 1 (HMGB1) were highly upregulated in premalignant keratinocytes after calcipotriol-plus–5-FU treatment (Figure 1, N–Q, and Supplemental Figure 3, H and I). Although human leukocyte antigen class I (HLA-I) expression was not affected by calcipotriol-plus–5-FU immunotherapy (Supplemental Figure 3, J and K), HLA-II was highly upregulated in AKs after calcipotriol-plus–5-FU treatment (Figure 1, R and S). HLA-II expression in AK keratinocytes was induced by calcipotriol-plus–5-FU therapy compared with 5-FU monotherapy (Supplemental Figure 3, L and M). The induction of HLA-II, DAMPs, and TSLP in premalignant keratinocytes after calcipotriol-plus–5-FU immunotherapy provides a robust axis for Th2 cell activation in AKs.

### T cell immunity induced by calcipotriol-plus–5-FU treatment persists over 5 years.

**Figure 2:**
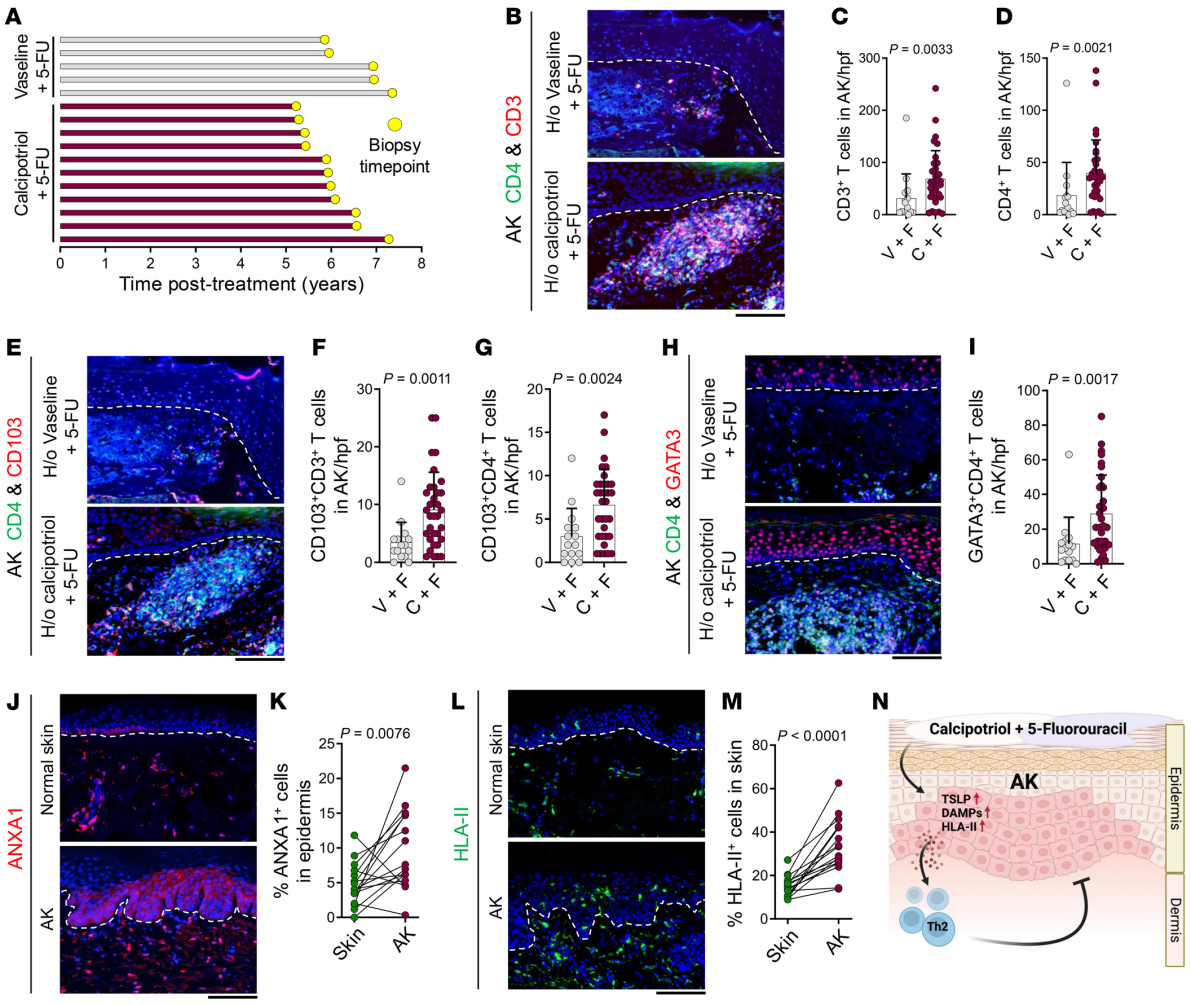
T cell immunity induced by calcipotriol-plus–5-FU immunotherapy persists over 5 years. (**A**) Time from Vaseline-plus–5-FU versus calcipotriol-plus–5-FU treatment to biopsy for each participant in the follow-up clinical study. (**B**) Representative images of CD4/CD3-stained AKs from participants who had a history of (H/o) Vaseline-plus–5-FU versus calcipotriol-plus–5-FU treatment. (**C** and **D**) Quantification of CD3^+^ T cells (**C**) and CD4^+^ T cells (**D**) in AKs from participants who had a history of Vaseline-plus–5-FU versus calcipotriol-plus–5-FU treatment. Each dot represents cell counts from a high power field (hpf) image. 3 hpf images are included per sample (*n* = 5 participants in Vaseline-plus–5-FU group, *n* = 11 participants in calcipotriol-plus–5-FU group, Mann-Whitney *U* test). (**E**) Representative images of CD4/CD103-stained AKs from participants who had a history of Vaseline-plus–5-FU versus calcipotriol-plus–5-FU treatment. (**F** and **G**) Quantification of CD103^+^CD3^+^ T cells (**F**) and CD103^+^CD4^+^ T cells (**G**) in AKs from participants who had a history of Vaseline-plus–5-FU versus calcipotriol-plus–5-FU treatment. Each dot represents cell counts from an hpf image. 3 hpf images are included per sample (*n* = 5 participants in Vaseline-plus–5-FU group, *n* = 11 participants in calcipotriol-plus–5-FU group, Mann-Whitney *U* test). (**H**) Representative images of CD4/GATA3-stained AKs from participants who had a history of Vaseline-plus–5-FU versus calcipotriol-plus–5-FU treatment. (**I**) Quantification of GATA3^+^CD4^+^ T cells in AKs from participants who had a history of Vaseline-plus–5-FU versus calcipotriol-plus–5-FU treatment. Each dot represents cell counts from an hpf image. 3 hpf images are included per sample (*n* = 5 participants in Vaseline-plus–5-FU group, *n* = 11 participants in calcipotriol-plus–5-FU group, Mann-Whitney *U* test). (**J**) Representative images of ANXA1-stained AKs and normal skin. (**K**) Quantification of ANXA1^+^ cells in the epidermis of AKs and normal skin. (*n* = 16 participants for AK and normal skin samples, paired *t* test). (**L**) Representative images of HLA-II-stained AKs and normal skin. (**M**) Quantification of HLA-II^+^ cells in AKs and normal skin. (*n* = 16 participants for AK and normal skin samples, paired *t* test). (**N**) Schematic diagram depicting the mechanism by which calcipotriol-plus–5-FU immunotherapy induces Th2 immunity against AKs. Bar graphs show mean + SD. Dashed lines mark the epidermal basement membrane in immunofluorescence images. Scale bars: 100 μm.

We have shown that calcipotriol-plus–5-FU immunotherapy lowers the risk of SCC development within 3 years after treatment (13). To determine whether T cell immunity induced by calcipotriol-plus–5-FU immunotherapy against AKs persists long-term, we collected AK and normal skin biopsies from the participants in the randomized trial comparing calcipotriol-plus–5-FU versus Vaseline-plus–5-FU for AK treatment over 5 years after the completion of the trial (9). We collected 5 pairs of AK and normal skin biopsies from participants who had a history of Vaseline-plus–5-FU treatment in the randomized clinical trial and never received calcipotriol-plus–5-FU since (Figure 2A). We collected 11 pairs of AK and normal skin biopsies from participants who received calcipotriol-plus–5-FU treatment either in the randomized clinical trial or open-label trial but never since (Figure 2A). Significantly more CD3^+^ T, CD4^+^ T, CD103^+^CD3^+^ T_RM_, and CD103^+^CD4^+^CD3^+^ T_RM_ cells infiltrated AKs of the participants who had a history of calcipotriol-plus–5-FU treatment compared with the participants who had a history of Vaseline plus 5-FU treatment (Figure 2, B–G, and Supplemental Figure 4A). GATA3^+^CD4^+^ T cells were increased in AKs of the participants who had a history of calcipotriol-plus–5-FU treatment compared with the participants who had a history of Vaseline-plus–5-FU treatment (Figure 2, H and I). Increased T cell infiltration in AKs was not observed in the normal skin of participants with a history of calcipotriol-plus–5-FU treatment (Supplemental Figure 4, B–F). To investigate which immune factors could be responsible for CD4^+^ T cell activation in AKs that developed in participants with a history of calcipotriol-plus–5-FU treatment, we evaluated ANXA1 and HLA-II expression in AK versus normal skin. AK keratinocytes showed higher expression of ANXA1 and HLA-II compared with normal skin (Figure 2, J–M). These results suggest that T cell immunity, originally induced by calcipotriol-plus–5-FU treatment, can be activated during AK development long after the treatment is completed, which may explain the reduced risk of SCC observed in calcipotriol-plus–5-FU–treated patients (Figure 2N) (13).

### CD4^+^ T cells are required for the tumor protective effect of calcipotriol-plus–5-FU immunotherapy.

**Figure 3:**
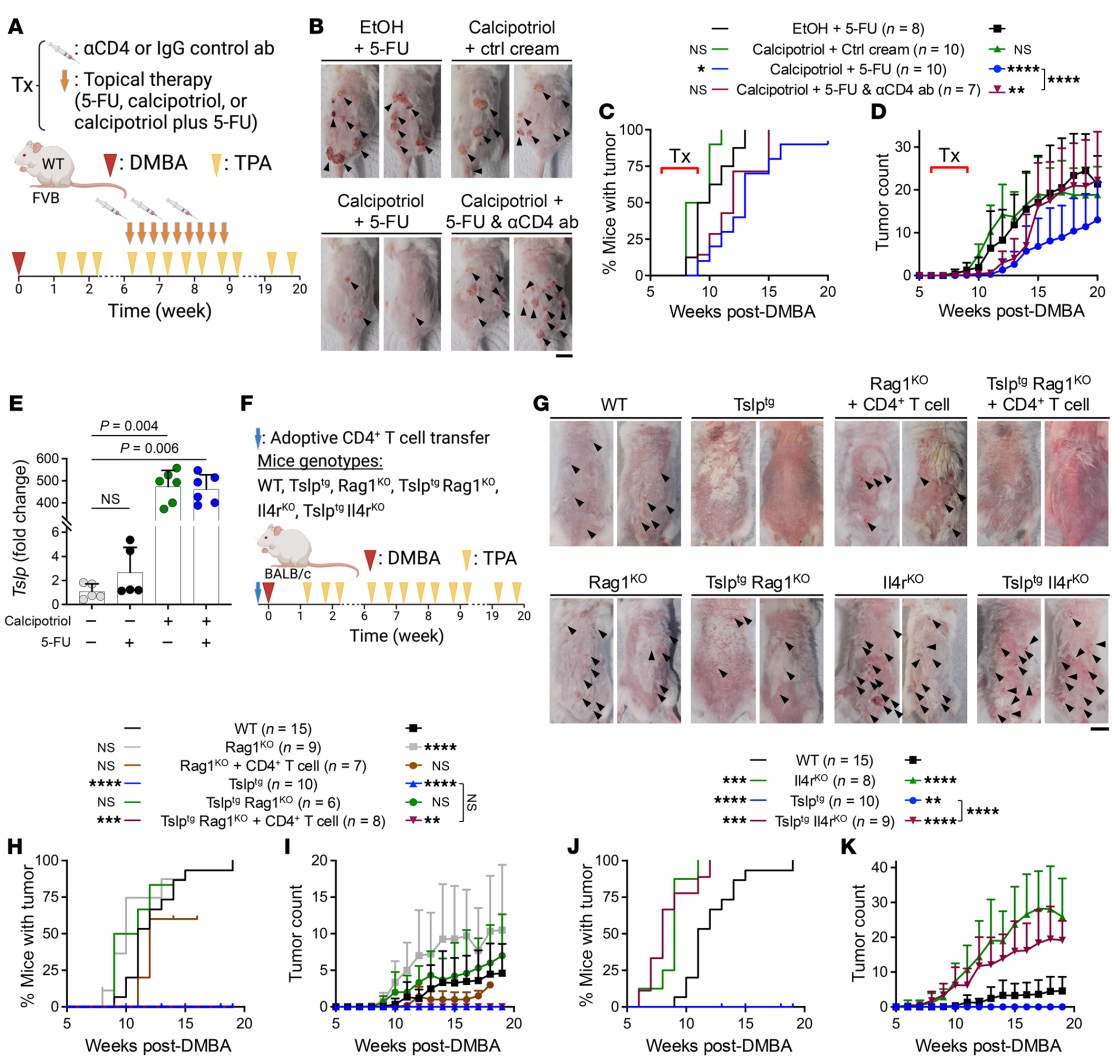
Calcipotriol-plus–5-FU immunotherapy prevents skin cancer development in a Th2 cell–dependent manner. (**A**) Schematic diagram of a 3-week topical therapy and CD4^+^ T cell depletion during the skin cancer development in WT mice on the FVB background undergoing DMBA/TPA skin carcinogenesis protocol. (**B**) Representative photographs of mouse back skin treated with EtOH-plus–5-FU, calcipotriol-plus–control cream, calcipotriol-plus–5-FU, and calcipotriol-plus–5-FU combined with anti-CD4 (α-CD4) antibody at week 20 after DMBA. Black arrows point to skin tumors. (**C** and **D**) Time to skin tumor onset (**C**, log-rank test) and the number of tumors per mouse over time (**D**, 2-way ANOVA with Dunnett’s multiple comparison test) in WT mice treated (Tx) with EtOH-plus–5-FU, calcipotriol-plus–control cream, calcipotriol-plus–5-FU, and calcipotriol-plus–5-FU combined with α-CD4 antibody. All groups are compared with EtOH-plus–5-FU group unless otherwise indicated. (**E**) Quantification of *Tslp* mRNA expression in murine skin treated with control vehicle, 5-FU, calcipotriol, or calcipotriol-plus–5-FU for 3 consecutive days. A day after last treatment, mRNA was isolated from the treated skin for analysis. Each dot represents a mouse (*n* = 5 in control vehicle and 5-FU groups, *n* = 6 in calcipotriol and calcipotriol-plus–5-FU groups, Kruskal-Wallis test with Dunn’s multiple comparison test). (**F**) Schematic diagram of adoptive T cell transfer and DMBA/TPA skin carcinogenesis in mice on the BALB/c background with different genotypes. (**G**) Representative photographs of mouse back skin at week 19 after DMBA. Black arrows point to skin tumors. (**H** and **I**) Time to tumor onset (**H**, log-rank test) and number of tumors per mouse over time (**I**, 2-way ANOVA with Dunnett’s multiple comparison test) in DMBA/TPA-treated WT, Rag1^KO^, Rag1^KO^ + CD4^+^ T cell, Tslp^tg^, Tslp^tg^ Rag1^KO^, and Tslp^tg^ Rag1^KO^ + CD4^+^ T cell groups. All groups are compared with the WT group. (**J** and **K**) Time to tumor onset (**J**, log-rank test) and number of tumors per mouse over time (**K**, 2-way ANOVA with Dunnett’s multiple comparison test) in DMBA/TPA-treated WT, Il4r^KO^, Tslp^tg^, and Tslp^tg^ Il4r^KO^ mice. All groups are compared with the WT group unless otherwise indicated. Please note that WT and Tslp^tg^ groups are common in **H**–**K**. Bar graphs show mean + SD. Scale bars: 1 cm. ****P < 0.0001, ***P < 0.001, **P < 0.01, *P < 0.05.

To determine the role of CD4^+^ T cells in mediating the efficacy of calcipotriol-plus–5-FU immunotherapy for skin cancer suppression in vivo, we studied the spontaneous chemical skin carcinogenesis model in mice (21). WT mice received 7,12-dimethylbenz(a)anthracene (DMBA) once on the back skin, followed by 12-O-tetradecanoyl-phorbol-13-acetate (TPA) application twice a week for 20 weeks to induce skin tumor development (Figure 3A). Topical EtOH-plus–0.5% 5-FU cream, 20 nmol calcipotriol-plus–control cream, or 20 nmol calcipotriol-plus–0.5% 5-FU treatment was applied to animals’ back skin 3 times a week from week 6 to 9 after DMBA (Figure 3A). Notably, calcipotriol-plus–5-FU treatment led to significantly delayed tumor onset and reduced tumor counts over time compared with EtOH-plus–5-FU and calcipotriol-plus–control cream treatment (Figure 3, B–D). CD4^+^ T cell depletion during topical calcipotriol-plus–5-FU treatment significantly diminished the efficacy of calcipotriol-plus–5-FU immunotherapy in WT mice (Figure 3, B–D, and Supplemental Figure 5A). These findings demonstrate that topical calcipotriol-plus–5-FU immunotherapy efficacy for skin cancer prevention depends on CD4^+^ T cell activation.

### CD4^+^ T cells are responsible for TSLP-mediated tumor protection in the skin.

Topical calcipotriol treatment alone or in combination with 5-FU induced TSLP expression in the skin (Figure 3E) (12, 22, 23). We have previously shown that the antitumor function of calcipotriol is TSLP dependent (9, 24–27). To investigate the mechanism by which TSLP induction in the skin leads to tumor protection, we subjected *K14-Tslp^tg/+^* (Tslp^tg^) mice that overexpress TSLP in skin keratinocytes to a skin carcinogenesis protocol (Figure 3F). DMBA/TPA-treated Tslp^tg^ mice did not develop skin tumors (Figure 3, G–I) (21, 24–28). Tslp^tg^ mice showed robust CD4^+^ T cell infiltration in the back skin at the completion of the chemical skin carcinogenesis protocol (Supplemental Figure 5, B and C). To investigate the role of CD4^+^ T cells in TSLP-mediated tumor protection, we transferred naïve CD4^+^ T cells from WT mice into *Rag1^–/–^* (Rag1^KO^) mice with or without TSLP overexpression. Without mature T and B cells, TSLP overexpression did not impact skin tumor development in Tslp^tg^ Rag1^KO^ compared with Rag1^KO^ and WT mice (Figure 3, G–I). However, CD4^+^ T cell transfer into Tslp^tg^ Rag1^KO^ reconstituted skin tumor protection in these animals (Figure 3, G–I). In contrast, Rag1^KO^ mice that received CD4^+^ T cells did not gain protection against skin tumor development (Figure 3, G–I). These findings demonstrate that CD4^+^ T cells are sufficient to mediate the TSLP-induced tumor protection in the skin even in the absence of CD8^+^ T and B cells.

### Th2 polarization is required for TSLP-mediated tumor protection.

To determine whether Th2 polarization was required in TSLP-activated CD4^+^ T cell immunity against skin carcinogenesis, we examined skin tumor development in *Il4ra^–/–^* (Il4r^KO^) and Tslp^tg^ Il4r^KO^ mice. IL-4/IL-4Rα signaling is required to differentiate and maintain Th2 CD4^+^ T cells (29, 30). TSLP overexpression induced GATA3^+^ Th2 polarization of CD4^+^ T cells, and mice that lacked IL-4Rα could not mount a Th2 immunity (Supplemental Figure 5, D and E). Mice lacking IL-4Rα showed earlier skin tumor onset and higher tumor counts over time compared with mice with intact IL-4Rα, regardless of TSLP expression (Figure 3, G, J, and K). To further investigate the role of direct TSLP signaling to CD4^+^ T cells in the establishment of TSLP-mediated tumor protection in the skin, we utilized CD4^+^ T cell transfer to Tslp^tg^ *Tslpr^–/–^* (Tslpr^KO^) animals. WT CD4^+^ T cell transfer reconstituted skin tumor protection in Tslp^tg^ Tslpr^KO^ mice, as shown by delayed tumor development compared with Tslp^tg^ Tslpr^KO^ mice that received Tslpr^KO^ CD4^+^ T cells (Supplemental Figure 5, F–H). Collectively, these findings demonstrate that Th2 polarization induced by TSLP signaling to CD4^+^ T cells is essential for TSLP-mediated antitumor immunity in the skin.

### Toxic autophagy and anoikis followed by apoptosis constitute the cell death mechanism induced by calcipotriol-plus–5-FU immunotherapy in AKs.

**Figure 4:**
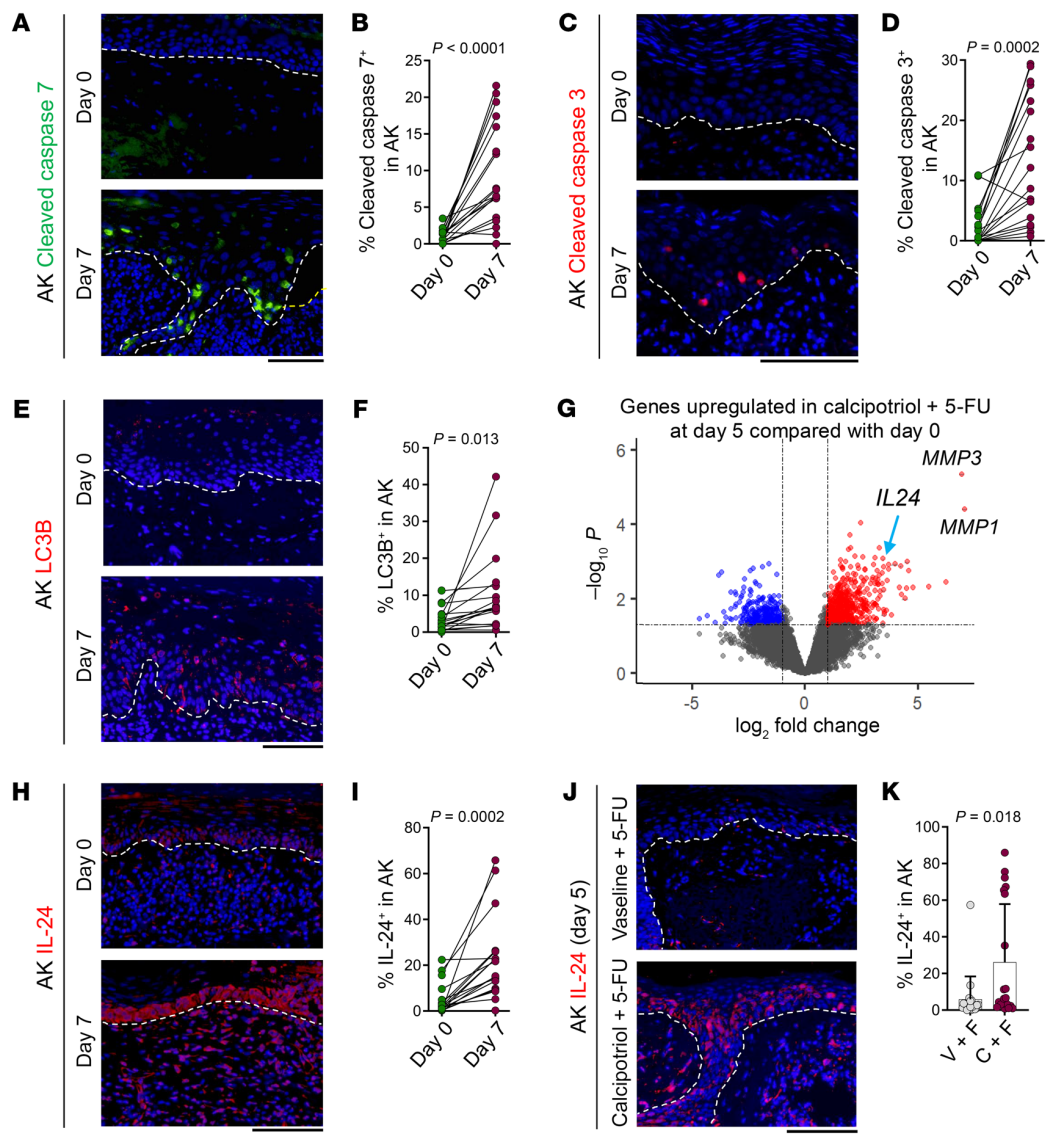
Calcipotriol-plus–5-FU treatment causes toxic autophagy and apoptosis associated with IL-24 induction in AK keratinocytes. (**A**) Representative images of cleaved caspase 7–stained AKs before (day 0) and after (day 7) calcipotriol-plus–5-FU treatment. (**B**) Quantification of cleaved caspase 7^+^ cells per total DAPI^+^ keratinocytes in AK before (day 0) and after (day 7) calcipotriol-plus–5-FU treatment. Each dot represents an AK sample (*n* = 18 participants at each time point, paired *t* test). (**C**) Representative images of cleaved caspase 3-stained AKs before (day 0) and after (day 7) calcipotriol-plus–5-FU treatment. (**D**) Quantification of cleaved caspase 3^+^ cells per total DAPI^+^ keratinocytes in AK before (day 0) and after (day 7) calcipotriol-plus–5-FU treatment. Each dot represents an AK sample (*n* = 18 participants at each time point, paired *t* test). (**E**) Representative images of LC3B-stained AKs before (day 0) and after (day 7) calcipotriol-plus–5-FU treatment. (**F**) Quantification of LC3B^+^ cells per total DAPI^+^ keratinocytes in AK before (day 0) and after (day 7) calcipotriol-plus–5-FU treatment. Each dot represents an AK sample (*n* = 18 participants at each time point, paired *t* test). (**G**) Volcano plot showing significantly upregulated (red dots) and downregulated (blue dots) genes in AKs collected from randomized clinical trial participants after calcipotriol-plus–5-FU treatment (day 5, *n* = 3) compared with before calcipotriol-plus–5-FU treatment (day 0, *n* = 3). Genes of interest are indicated with their symbol. (**H**) Representative images of IL-24–stained AKs before (day 0) and after (day 7) calcipotriol-plus–5-FU treatment. (**I**) Quantification of IL-24^+^ cells per total DAPI^+^ keratinocytes in AK before (day 0) and after (day 7) calcipotriol-plus–5-FU treatment. Each dot represents an AK sample (*n* = 18 participants at each time point, paired *t* test). (**J**) Representative images of IL-24–stained AKs treated with Vaseline-plus–5-FU versus calcipotriol-plus–5-FU (day 5). (**K**) Quantification of IL-24^+^ cells per total DAPI^+^ keratinocytes in AK treated with Vaseline-plus–5-FU (V + F) and calcipotriol-plus–5-FU (**C** and **F**). Each dot represents an AK sample (*n* = 20 participants for both treatment groups, Mann-Whitney *U* test). Bar graphs show mean + SD. Dashed lines mark the epidermal basement membrane in immunofluorescence images. Scale bars: 100 μm.

To determine the mechanism of AK clearance in response to calcipotriol-plus–5-FU immunotherapy, we investigated the expression of major cell death pathways, including apoptosis (cleaved caspase-7 and cleaved caspase-3 as markers), pyroptosis (cleaved gasdermin D as a marker), and toxic autophagy (light chain 3 β or LC3B as a marker). Cleaved caspase-7, cleaved caspase-3, and LC3B expression were induced by calcipotriol-plus–5-FU immunotherapy (Figure 4, A–F). By contrast, cleaved gasdermin D was not upregulated in AK keratinocytes after calcipotriol-plus–5-FU immunotherapy (Supplemental Figure 6A). Notably, we consistently observed histological detachment of AK keratinocytes from the basement membrane after calcipotriol-plus–5-FU immunotherapy, which is the hallmark of anoikis, the process of epithelial cell detachment from the extracellular matrix accompanied by activation of caspases and apoptosis (Figure 1C) (31–35). These results indicate that premalignant keratinocytes undergo toxic autophagy and anoikis, resulting in apoptosis in response to calcipotriol-plus–5-FU immunotherapy.

### Effector mechanism mediating the premalignant keratinocyte death by calcipotriol-plus–5-FU immunotherapy.

To explore the effector mechanism leading to cell death in response to calcipotriol-plus–5-FU immunotherapy, we compared the transcriptome of AKs treated with calcipotriol-plus–5-FU with (a) AKs before treatment and (b) AKs after Vaseline-plus–5-FU treatment using samples collected in our randomized clinical trial (9). *IL24*, *MMP1*, and *MMP3* were among the top 10 upregulated genes in both comparisons (Figure 4G and Supplemental Table 2). IL-24 induces toxic autophagy and apoptosis in a cancer-selective manner (14, 15, 17, 36–38). MMPs are implicated in the remodeling of integrins, whose loss can cause anoikis (39, 40). We confirmed the upregulation of IL-24 and MMP1 in AK keratinocytes after calcipotriol-plus–5-FU immunotherapy compared with before treatment and Vaseline plus 5-FU treatment (Figure 4, H–K, and Supplemental Figure 6, B and C). IL-24 expression was not altered in normal skin treated with calcipotriol-plus–5-FU (Supplemental Figure 6, D and E).

### IL-24 is induced by Th2-associated cytokines in cancer cells and its overexpression causes toxic autophagy in the cells.

**Figure 5:**
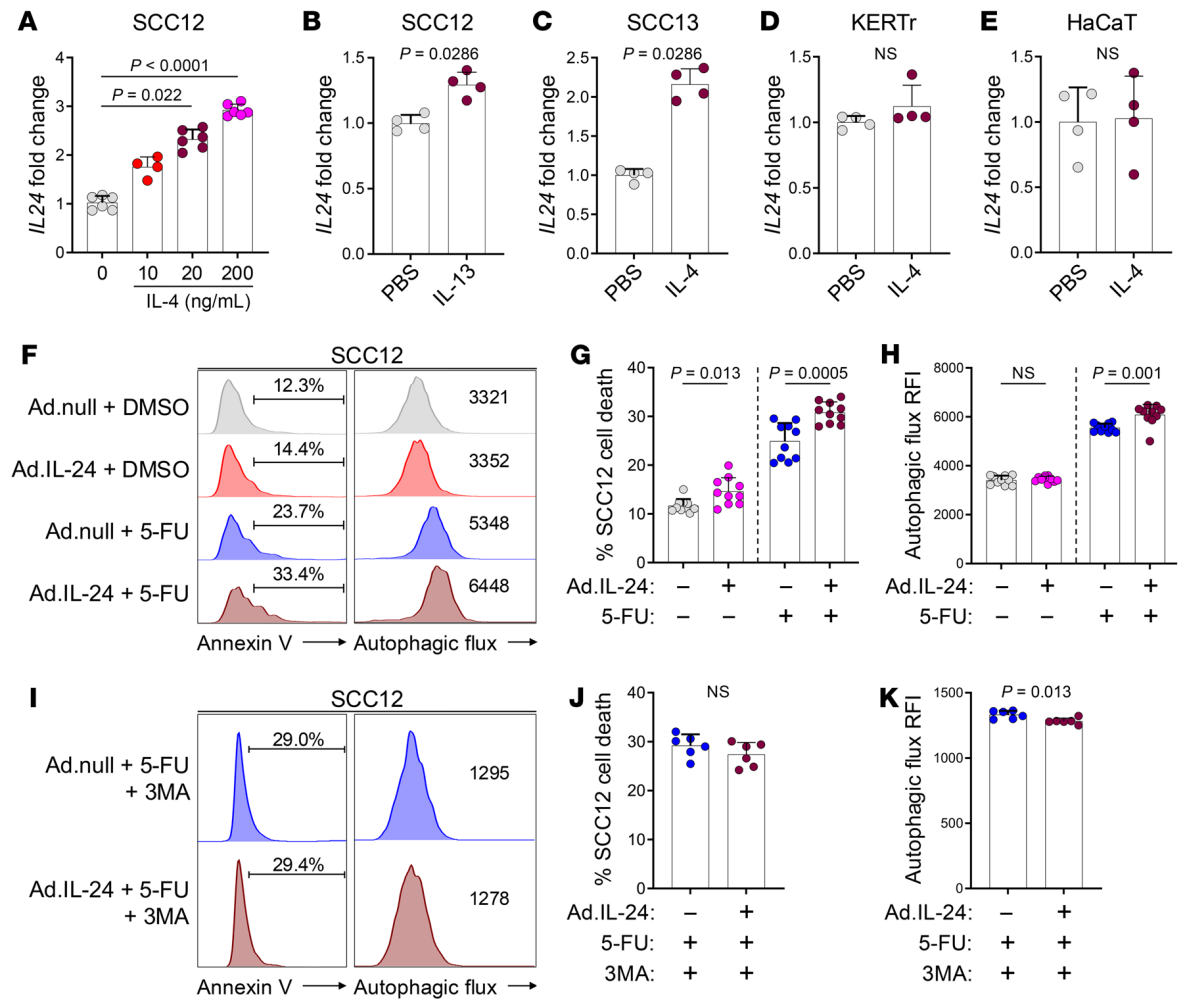
IL-24 is induced by Th2-associated cytokines in cancer cells, and its overexpression causes toxic autophagy in the cells. (**A**) Quantification of *IL24* mRNA expression in SCC12 cells treated with IL-4 for 4 hours. Each dot represents a biological replicate (0, 20, 200 ng/mL: *n* = 6, 10 ng/mL: *n* = 4, Kruskal-Wallis test with Dunn’s multiple comparison test). (**B**) Quantification of *IL24* mRNA expression in SCC12 cells treated with 20 ng/mL of IL-13 for 4 hours. Each dot represents a biological replicate (*n* = 4 in each group, Mann-Whitney *U* test). (**C**) Quantification of *IL24* mRNA expression in SCC13 cells treated with 20 ng/mL of IL-4 for 4 hours. Each dot represents a biological replicate (*n* = 4 in each group, Mann-Whitney *U* test). (**D** and **E**) Quantification of *IL24* mRNA expression in KERTr (**D**) and HaCaT (**E**) normal keratinocytes treated with 20 ng/mL of IL-4 for 4 hours. Each dot represents a biological replicate (*n* = 4 in each group, Mann-Whitney *U* test). (**F**–**H**) Representative flow cytometry histogram of annexin V and autophagic flux (**F**), and quantification of percentage cell death (**G**) and RFI of autophagic flux (**H**) in SCC12 cells infected with adenovirus vector overexpressing IL-24 (Ad.IL-24) and treated with 5-FU compared with control vector (Ad.null) and DMSO (*n* = 10 in each group, Mann-Whitney *U* test). (**I**–**K**) Representative flow cytometry histogram of annexin V and autophagic flux (**I**), and quantification of percentage cell death (**J**) and RFI of autophagic flux (**K**) in SCC12 cells infected with Ad.IL-24 and treated with 5-FU in the presence of 3MA compared with Ad.null (*n* = 6 in each group, Mann-Whitney *U* test). Bar graphs show mean + SD.

Next, we investigated whether Th2-associated cytokines IL-4 and IL-13 (41) induced IL-24 expression in malignant keratinocytes. IL-4 and IL-13 induced *IL24* expression in SCC cells in a dose-dependent manner (Figure 5, A–C). By contrast, IL-4 did not alter *IL24* expression in normal keratinocyte cell lines KERTr and HaCaT (Figure 5, D and E). SCC cells expressed IL-4 receptor α, IL-13 receptor α1, and γc receptor, while IL-4 receptor α was not expressed on the surface of normal keratinocyte cell lines (Supplemental Figure 7, A–C).

To test whether IL-24 induction can kill malignant keratinocytes in combination with 5-FU, we infected SCC12 cells with Ad.IL-24, an adenoviral vector overexpressing IL-24 (15, 37, 42–49), and treated the cells with 5-FU in vitro. Ad.IL-24-plus–5-FU caused more SCC cell death and autophagic flux compared with Ad.null-plus–5-FU (empty vector control with 5-FU) (Figure 5, F–H, and Supplemental Figure 7, D and E). By contrast, Ad.IL-24 treatment alone did not induce autophagic flux in SCC cells (Figure 5, F–H, and Supplemental Figure 7, D and E). In addition, cell death and autophagy induction were not observed in normal keratinocyte cell line treated with Ad.IL-24-plus–5-FU (Supplemental Figure 7, F–H). Ad.IL-24-plus–5-FU–induced cell death and autophagy were inhibited by 3-methyladenine (3MA), an autophagy inhibitor (Figure 5, I–K) (50). These results suggest that IL-24 induction by calcipotriol-activated Th2 cells synergizes with 5-FU to cause AK keratinocyte death through toxic autophagy.

### Calcipotriol-plus–5-FU–mediated skin cancer prevention is IL-24–dependent.

**Figure 6:**
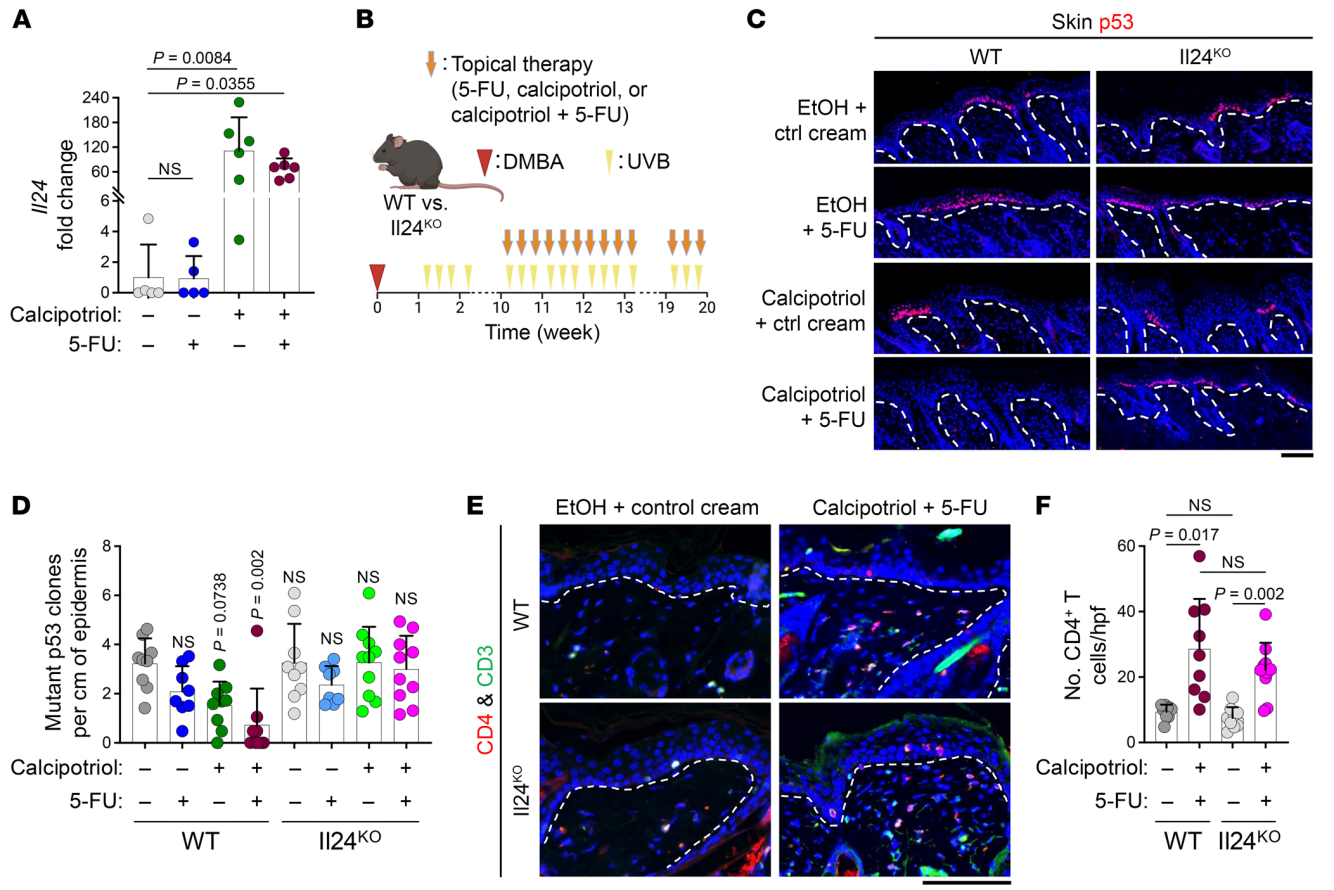
Calcipotriol-plus–5-FU immunotherapy–mediated skin cancer protection is IL-24–dependent. (**A**) Quantification of *Il24* mRNA expression in murine skin treated with control vehicle, 5-FU, calcipotriol, or calcipotriol-plus–5-FU for 3 consecutive days. 1 day after the last treatment, mRNA was isolated from the treated skin. Each dot represents a mouse (*n* = 5 in the control and 5-FU groups, *n* = 6 in calcipotriol and calcipotriol-plus–5-FU groups, Kruskal-Wallis test with Dunn’s multiple comparison test). (**B**) Schematic diagram of a 10-week topical therapy during skin cancer development in mice on the C57BL/6 background exposed to DMBA/UVB skin carcinogenesis protocol. (**C**) Representative images of p53-stained back skin of WT and Il24^KO^ mice treated with EtOH-plus–control cream, EtOH-plus–5-FU, calcipotriol-plus–control cream, or calcipotriol-plus–5-FU at week 20 after DMBA. Note that mutant p53 clones in the epidermis are marked by increased p53 protein levels in the mutant cells. (**D**) Quantification of mutant p53 clones per cm of the epidermis in the back skin of WT and Il24^KO^ mice treated with EtOH-plus–control cream, EtOH-plus–5-FU, calcipotriol-plus–control cream, or calcipotriol-plus–5-FU at week 20 after DMBA. Each dot represents a mouse (WT EtOH-plus–control cream: *n* = 9, WT EtOH-plus–5-FU: *n* = 8, WT calcipotriol-plus–control cream: *n* = 9, WT calcipotriol-plus–5-FU: *n* = 9, Il24^KO^ EtOH-plus–control cream: *n* = 9, Il24^KO^ EtOH-plus–5-FU: *n* = 8, Il24^KO^ calcipotriol-plus–control cream: *n* = 10, Il24^KO^ calcipotriol-plus–5-FU: *n* = 10, Kruskal-Wallis test with Dunn’s multiple comparison test). (**E**) Representative images of CD4/CD3-stained back skin of WT and Il24^KO^ mice treated with EtOH-plus–control cream versus calcipotriol-plus–5-FU at week 20 after DMBA. (**F**) Quantification of CD4^+^ T cells in the back skin of WT and Il24^KO^ mice treated with control vehicle versus calcipotriol-plus–5-FU at week 20 after DMBA. Each dot represents a mouse (WT EtOH-plus–control cream: *n* = 9, WT calcipotriol-plus–5-FU: *n* = 9, Il24^KO^ EtOH-plus–control cream: *n* = 9, Il24^KO^ calcipotriol-plus–5-FU: *n* = 10, Kruskal-Wallis test with Dunn’s multiple comparison test). Bar graphs show mean + SD. Dashed lines mark the epidermal basement membrane in immunofluorescence images. Scale bars: 100 μm.

Topical calcipotriol alone or in combination with 5-FU upregulated *Il24* expression in mouse skin (Figure 6A). Likewise, TSLP overexpression induced *Il24* expression in the skin (Supplemental Figure 8A). To establish a platform to investigate the effects of calcipotriol-plus–5-FU immunotherapy on skin cancer precursors in vivo, we examined the efficacy of calcipotriol-plus–5-FU immunotherapy in controlling mutant p53 clones in the DMBA-plus–ultraviolet radiation (DMBA-plus–UVB) skin carcinogenesis model (51). AKs express mutated p53 (52), and the development of mutant p53 clones in the DMBA/UVB skin carcinogenesis model mimics how AK develops in humans (52–56). Calcipotriol-plus–5-FU immunotherapy reduced the number of UVB-induced mutant p53 clones in the skin of hairless immunocompetent SKH-1 mice compared with 5-FU monotherapy (Supplemental Figure 8, B and C) (57). To investigate whether IL-24 is required for the tumor protective effect of calcipotriol-plus–5-FU immunotherapy, we compared mutant p53 clone development in WT and *Il24^–/–^* (Il24^KO^) mice on the C57BL/6 background, which received control, 5-FU, calcipotriol, or calcipotriol-plus–5-FU treatment (Figure 6B). Calcipotriol-plus–5-FU treatment reduced the number of mutant p53 clones in the epidermis compared with the control treatment in WT mice (Figure 6, C and D). Calcipotriol monotherapy also showed a trend toward reduction in mutant p53 clones in WT mice (Figure 6, C and D). However, calcipotriol-plus–5-FU treatment did not reduce the number of mutant p53 clones in Il24^KO^ animals (Figure 6, C and D). Importantly, calcipotriol-plus–5-FU treatment’s ability to induce CD4^+^ T cell response in the skin was not affected by the loss of IL-24 (Figure 6, E and F). These results indicate that IL-24 is a critical effector molecule downstream of CD4^+^ T cell activation by calcipotriol-plus–5-FU immunotherapy, which is required to eliminate precancerous skin lesions.

## Discussion

Our findings demonstrate that calcipotriol-plus–5-FU–stimulated Th2 immunity eliminates precancerous skin lesions by inducing IL-24 in premalignant keratinocytes, which causes toxic autophagy in a cancer-selective manner (14, 15, 17, 38). Calcipotriol-plus–5-FU immunotherapy heightens AK immunogenicity by upregulating TSLP, DAMPs, and HLA-II expression in premalignant keratinocytes, culminating in a robust Th2 immunity against AKs. Our follow-up clinical study suggests that calcipotriol-plus–5-FU–mediated Th2 immunity persists for years after treatment and is reactivated during AK development, which may suppress its progression to SCC. CD4^+^ Th2 cells are necessary to establish tumor protection in response to TSLP in the skin, and this protective effect persists in the absence of CD8^+^ T or B cells. Th2 cell effector function is mediated at least in part by the type 2 cytokines IL-4 and IL-13, inducing IL-24 in transformed keratinocytes. Together, our findings substantiate the role of antitumor Th2 cell immunity in early cancer development, which can be leveraged for cancer immunoprevention.

The discovery of Th2 cells as mediators of antitumor immunity against skin cancer precursors in humans provides fundamental insights into the importance of Th2 cells in cancer immunosurveillance. In protumorigenic immune microenvironments, Th2 cells exhibit elevated levels of cytokines such as IL-10 and TGF-β and contribute to tumor progression (58). By contrast, inflammatory Th2 cells induced by TSLP effectively protect epithelial cells against malignant transformation (24–28, 59, 60). Furthermore, we have recently demonstrated that TSLP-activated Th2 cells directly block breast cancer development by terminally differentiating the tumor cells to form gland-like structures (24). CD4^+^ T cell induction for cancer immunoprevention and therapy has several distinct advantages compared with conventional immunotherapies directed at CD8^+^ T cells. CD4^+^ T cells are initiators of adaptive immunity, and their direct activation to target tumor antigens can launch a robust antitumor immune response in early epithelial cancers and precancerous lesions (61).

We have identified IL-24 as a downstream effector molecule induced by Th2 immunity in transformed keratinocytes. IL-24 was first identified by subtraction hybridization from terminally differentiating metastasis-derived human melanoma cells (36). Mounting evidence supports the role of IL-24, or melanoma differentiation–associated gene-7 (14, 15, 17, 36), in inducing toxic autophagy and apoptosis in solid cancers, including melanoma and epithelial cancers (42, 43, 48, 49, 62–66). IL-24 induces apoptosis through a combination of intracellular and secretory mechanisms (14, 15, 17, 42, 67). Although our findings point to intracellular IL-24–mediated cell death induction, we cannot exclude the possibility that secreted IL-24 contributes to toxic autophagy and apoptosis through its receptor on premalignant keratinocytes. In support of this possibility, a significant contributor to the antitumor properties of IL-24 is its ability to be secreted, promoting potent “bystander” anticancer properties (14–17, 37, 42, 43, 68). Moreover, transgenic animal models of breast cancer involving production and secretion of IL-24 by the mouse mammary gland have documented protective effects against breast cancer development (49). Cancer immunotherapy is enhanced by delivering IL-24 in genetically engineered T cells, further supporting its potential as a safe anticancer cytokine (69). Calcipotriol-plus–5-FU immunotherapy induces DAMPs, which are implicated in ER stress (70). ER stress renders cancer cells more vulnerable to IL-24–mediated toxic effects (14–17, 37, 62, 68, 69, 71–77). Anoikis is another cell death mechanism, which is caused by loss of adhesion (78). Unlike the broad presence of apoptotic and necrotic keratinocytes at the site of epidermal detachment in toxic epidermal necrolysis (79), a significant portion of AK basal keratinocytes at the site of epidermal detachment after calcipotriol-plus–5-FU treatment did not show signs of apoptosis. This observation suggests that epidermal detachment preceding apoptosis (i.e., anoikis) contributes to AK clearance by calcipotriol-plus–5-FU treatment. Thus, calcipotriol-plus–5-FU immunotherapy causes AK keratinocyte death by inducing toxic autophagy and anoikis, culminating in apoptosis. This mechanism of action is distinct from common perforin/granzyme-based cytotoxicity induced by current immunotherapies (80). Whether toxic autophagy and anoikis synergize to cause cell death in AK keratinocytes warrants future investigation.

The efficacy of transient TSLP induction in delivering lasting tumor-specific immunity in the skin emphasizes the benefit of using topical TSLP inducers (e.g., calcipotriol) as safe and accessible modalities for skin cancer immunoprevention. We previously reported that a 4-day calcipotriol-plus–5-FU treatment on the face and scalp leads to the induction of robust erythema, T cell immunity, and T_RM_ formation against AKs and significantly lowers SCC risk within 3 years after treatment compared with 5-FU monotherapy (9, 13). Here, we further characterize the robust T cell immunity against AKs newly developed in patients with a distant history of calcipotriol-plus–5-FU treatment. The long-term heightened T cell response in AKs developed after calcipotriol-plus–5-FU immunotherapy substantiates its efficacy in preventing SCC (13). Therefore, inducing Th2 cell immunity in AKs may improve clinical outcomes for patients with skin cancer by preventing the development of invasive SCC.

Although calcipotriol is a well-established topical inducer of TSLP in mouse skin (12, 22, 23), it has been reported that calcipotriol does not induce TSLP in normal human skin (19). We show that calcipotriol-plus–5-FU immunotherapy induces TSLP expression preferentially in AKs compared with normal skin. We find that 1 μΜ calcipotriol induces TSLP expression in SCC cells but not in normal human keratinocyte cell lines, which is consistent with previous reports (19, 81). Consistently, calcipotriol-plus–5-FU immunotherapy induces Th2 immunity in AKs but not in normal human skin (9). Thus, topical calcipotriol therapy in humans preferentially targets transformed keratinocytes for TSLP expression, adding to the specificity of calcipotriol-plus–5-FU field therapy for immune induction in AKs while avoiding a generalized inflammatory response in normal skin.

In summary, our findings establish the previously unrecognized role of Th2 immunity against premalignant lesions in humans. Th2 cells activated by topical TSLP, DAMPs, and HLA-II induction in AK keratinocytes cause toxic autophagy and apoptosis in AKs. Once T cell immunity eliminates premalignant keratinocytes, T_RM_ cells can persist and protect the skin from cancer development. Our work highlights the importance of the immune-activating signals released by epithelial cells as upstream activators of antitumor immunity, which can be therapeutically leveraged for cancer immunoprevention and treatment.

## Methods

### Sex as a biological variable.

Our study examined male and female mice, and similar findings were reported for both sexes.

### Clinical studies.

We performed an open-label clinical trial in the participants of the calcipotriol-plus–5-FU versus Vaseline-plus–5-FU randomized clinical trial (NCT02019355). All participants were immunocompetent. Eighteen participants with persistent AKs after the treatment in the randomized trial were enrolled in the open-label trial. All participants received a topical preparation of 0.005% calcipotriol ointment (Taro Pharmaceuticals) combined with 5% 5-FU cream (Taro Pharmaceuticals) premixed at 1:1 weight ratio (final concentration: 0.0025% calcipotriol and 2.5% 5-FU) for twice-daily 6-day self application (Supplemental Figure 1, A and B). AKs were clinically defined as pink, scaly papules on sun-damaged skin. A 6-day treatment duration was chosen to heighten immune induction by calcipotriol-plus–5-FU treatment while avoiding severe side effects (9). The drug combinations were prepared according to the United States Pharmacopeial Convention 795 (USP 795) guidelines for compounding topical medications and under the supervision of the investigational drug pharmacies of Washington University in St. Louis. The trial was conducted at Washington University Medical Center between September 2014 and May 2015. Inclusion criteria included age of at least 50 years and the presence of 4 to 15 clinically visible and discrete AKs in a 25-cm^2^ contiguous area on any of the 4 anatomical sites (Supplemental Figure 1A). Exclusion criteria included immunosuppression and a recent (within 1 month) use of medications that could hinder assessment of the treated skin. If a participant had 2 or more qualified anatomical sites, 1 was selected as the primary anatomical site. Participants’ written consents were obtained. At the initial visit (day 0), AK numbers and anatomical location were documented and photographed, and the study medications were dispensed. Participants applied the study medication to the entirety of their qualified anatomical sites twice daily for 6 consecutive days, starting the day after their first visit. Participants underwent evaluation on day 7 and at week 8 (Supplemental Figure 1B). Skin biopsies were obtained before (day 0) and after treatment (day 7). Biopsies were performed on a randomly selected AK and adjacent normal skin from each participant at each time point by a trained dermatologist. For the follow-up clinical sample collection, the participants in the randomized trial were seen in clinic for a randomly selected AK and adjacent normal skin biopsy from the anatomical sites previously treated with calcipotriol-plus–5-FU versus Vaseline-plus–5-FU.

### Cell lines.

James G. Rheinwald (Brigham and Women’s Hospital, Boston, Massachusetts, USA) provided human skin SCC cell lines, SCC12 and SCC13 (82, 83) and N/TERT-1 (84). KERTr, a normal human keratinocyte cell line, was purchased from ATCC (CRL-2309). Anna Mandinova (Massachusetts General Hospital, Boston, Massachusetts, USA) provided HaCaT, a normal human keratinocyte cell line. SCC12, SCC13, and HaCaT were cultured in DMEM medium (Thermo Fisher Scientific), including 10% FBS, 1% penicillin/streptomycin, and 1% glutamine (Sigma-Aldrich), at 37°C in 5% CO_2_. KERTr and N/TERT-1 were cultured in Keratinocyte-SFM media including bovine pituitary extract, recombinant epidermal growth factor (Thermo Fisher Scientific) and 1% penicillin/streptomycin, at 37°C in 5% CO_2_.

### Histology.

Tissue samples were fixed with 4% paraformaldehyde (PFA) and embedded in paraffin. Sections of 5 μm were cut and deparaffinized. After being permeabilized with 0.2% Triton-X (Thermo Fisher Scientific) in PBS for 5 minutes, antigen retrieval was performed using a pressure cooker in citrate-based antigen unmasking solution (Vector Laboratories) or tris-based antigen unmasking solution (Vector Laboratories) for 20 minutes. Slides were rinsed once in DI water and in PBS, including 0.1% Tween 20 (Sigma-Aldrich) (PBS-T). Slides were blocked with 5% normal goat serum (Sigma-Aldrich) in PBS and incubated in a humidified chamber at room temperature for 1 hour. Slides were incubated overnight at 4°C with primary antibodies diluted in the blocking buffer (antibody information is included in Supplemental Table 3). When stained with 2 primary antibodies from the same host species, signal amplification and heat-induced stripping for antibodies were done following the standard protocol using Opal 4-Color IHC Kit (PerkinElmer). Following primary antibody application, slides were rinsed once and washed 3 times for 2 minutes each in PBS-T. Slides were incubated in secondary antibodies (Supplemental Table 3) and 4’,6-diamidino-2-phenylindole (DAPI, Invitrogen; 1:4000) diluted in the blocking buffer for 60 minutes at room temperature. Slides were washed in PBS-T and mounted with Fluoroshield (Sigma-Aldrich). The stained tissues were imaged with a ZEISS Axio Scan.Z1 Slide Scanner (Zeiss). For H&E staining, slides were stained according to standard procedures and mounted with Cytoseal XYL (Thermo Fisher Scientific). For frozen tissue, 7 μm sections were obtained. Sections were fixed in acetone at –20°C for 10 minutes. Slides were blocked with 5% normal goat serum in PBS without antigen retrieval. All the steps after blocking were the same as described above for formalin-fixed paraffin-embedded tissue sections. For chromogenic IHC staining, sections were incubated with primary antibodies (primary antibody information is available in Supplemental Table 3) for 16 minutes, followed by horseradish peroxidase–conjugated (HRP) secondary antibodies (ImmPRESS HRP Horse Anti-Rabbit IgG Polymer Detection Kit, Peroxidase, Vector Laboratories, MP-7401). Sections were stained either with Ventana’s Discovery ChromoMap DAB or Discovery Purple staining kits (Roche). Sections were counterstained with hematoxylin.

### Image analysis.

Automated counting was performed using Halo 3.0 software (Indica Labs). Cell counts were reported as percentage-positive cells per total DAPI-positive cells in AK keratinocytes and normal epidermal keratinocytes unless otherwise specified. Immune cell counts were reported as number of cells per high-power fields (hpf, 200× magnification) unless otherwise specified. Cell counts were done across up-to 10 randomly selected hpf for mouse tissue or up to 3 hpf for human tissue samples, depending on the tissue size.

### RNA-seq analysis.

AK biopsy samples collected from the randomized clinical trial were lysed with RNA Lysis Buffer and RNA was isolated following the protocol of Quick-RNA Miniprep Kit (Zymo Research) and quantified using Agilent Bioanalyzer 2100 (Agilent) (9). Libraries were prepared by Novogene using the NEBNext Ultra RNA Library Prep kit for Illumina (New England Biolabs). Sequencing was performed using the Illumina NovaSeq6000 System. Reads were aligned to the human reference genome (hg19) using STAR-2.5 (85). Transcript assembly and quantification were performed using StringTie-1.3.6 and Ballgown-2.0.0 (86, 87). Differential expression analysis was performed with limma-3.24.15 using count values (88). Batch effects among biological replicates were corrected using the lmFit, contrasts.fit, and eBayes functions in limma. *P* values were calculated using limma in R. log2 ratio > | 1| and *P* < 0.05 was considered significant. Original RNA-seq data are available in the NCBI Gene Expression Omnibus (GEO) with accession number GSE255479.

### Flow cytometry.

Cells were washed once with PBS, including 5% newborn calf serum (Thermo Fisher Scientific) and 0.01% sodium azide (Sigma-Aldrich), and stained with antibodies on ice for 30 minutes (Supplemental Table 3). Following the surface marker staining, cells were fixed and permeabilized for intracellular staining using Fixation Buffer (BioLegend) and Intracellular Staining Perm Wash Buffer (BioLegend) when an intracellular marker was evaluated. Permeabilized cells were stained with antibodies overnight at 4°C (Supplemental Table 3). For the cell death assay, staining was done following the standard protocol of Annexin V Apoptosis Detection Kit with PI (BioLegend). For autophagic flux assay, staining was done following the manufacturer’s instruction of CYTO-ID Autophagy detection kit (Enzo Biochem). Propidium iodide–positive cells were regarded as dead cells. Cells were washed and examined by BD LSRFortessa X-20 flow cytometer (BD Bioscience). Data were analyzed using FlowJo software (BD Bioscience).

### Quantitative PCR.

RNA was isolated following the Quick-RNA Miniprep Kit instructions (Zymo Research) and quantified using Nano-Drop ND-1100 (NanoDrop Technologies). cDNA samples were synthesized from 1 μg of total RNA using Invitrogen SuperScripts III Reverse Transcriptase (Thermo Fisher Scientific). Expression levels of all cDNA samples were determined with QuantStudio 5 (Thermo Fisher Scientific) using iTaq Universal SYBR green supermix (Bio-Rad Laboratories). Primer sequences for SYBR green assays are listed in Supplemental Table 3. Quantitative real-time PCR using SYBR green analyses were performed in a final reaction volume of 10 μL consisting of 2 μL of cDNA of the respective sample and 8 μL of SYBR green master mix mixed with the corresponding primers (2 μM) for each gene. All assays were conducted in triplicate and normalized to *GAPDH/Gapdh* expression.

### Cytokine and drug treatment in vitro.

Cells were treated with recombinant human IL-4 and IL-13 (BioLegend) at the concentration of 20 ng/mL for 4 hours in serum-reduced DMEM medium (Thermo Fisher Scientific), including 1% FBS to reduce analytical interference and provide more reproducible experimental conditions unless indicated otherwise (89). Cells were treated with 1 μM calcipotriol (Sigma-Aldrich) for 1 day in serum-reduced DMEM medium.

### Adenovirus transfection.

Ad.IL-24 and Ad.null (negative control) were kindly provided by Paul B. Fisher’s lab (37, 43, 48). Cells were seeded at 1.0 × 10^5^ cells/mL and 1 day after seeding, adenovirus was added in serum-free, antibiotic-free DMEM at 1,000 virus particles per cell. Cells were incubated for 2 hours while gently rocking every 15 minutes. 2 hours later, complete media containing FBS was added. Twenty-four hours later, 10 μM 5-FU or DMSO (carrier control) was added to the cells.

### Animal studies.

All mice were housed under pathogen-free conditions in an animal facility at Massachusetts General Hospital in accordance with animal care regulations. The following mouse lines were used in the study: WT C57BL/6J (Jackson Laboratory, catalog no. 000664), B6.Cg-Foxn1^nu^/J (B6 nude mice, Jackson Laboratory, catalog no. 000819), SKH-1 Elite (Charles River Laboratories, catalog no. 477), WT FVB (Charles River Laboratories, catalog no. 207), WT BALB/c (Charles River Laboratories, catalog no. 555), *K14-Tslp^tg^* (Tslp^tg^, a gift of Andrew Farr, University of Washington, Seattle, Washington, USA), *Tslpr^−/−^* (Tslpr^KO^, a gift of Warren Leonard, National Institutes of Health, Bethesda, Maryland, USA), *Il4ra^tm1Sz/J^* (Il4r^KO^, Jackson Laboratory, catalog no. 002518), C.129S7(B6)-Rag1^tm1Mom^/J (Rag1^KO^, Jackson Laboratory, catalog no. 003145) and C57BL/6N-*Il24^tm1.1(KOMP)Vlcg^/JMmucd* (IL24^KO^, Mutant Mouse Resource & Research Centers, catalog no. 048196-UCD). Tslp^tg^ mice were maintained on the BALB/c and C57BL/6 backgrounds. Rag1^KO^ or Il4r^KO^ mice were maintained on the BALB/c background. Il24^KO^ and Tslpr^KO^ mice were maintained on the C57BL/6 background. Mutant mice were genotyped using the primers listed in Supplemental Table 3. Age- and gender-matched female mice were used in all experiments.

### DMBA/TPA skin carcinogenesis.

Mice aged 8–10 weeks received 100 μg DMBA in 200 μL acetone on the back skin (Week 0). A week later, mice received topical application of 6 μg of Phorbol 12-myristate 13-acetate (TPA, Sigma-Aldrich) in acetone to the shaved back skin twice a week for 19 weeks. Mice were observed 3 times a week until the biggest tumor size reached 15 mm in diameter. Mice were harvested 20 weeks after DMBA.

### DMBA/UVB skin carcinogenesis.

Mice aged 8–10 weeks received 100 μg DMBA (Sigma-Aldrich) in 200 μL acetone on the back skin (Week 0). A week later, mice were exposed to 250 mJ/cm^2^ ultraviolet B (UV) 3 times per week for up to 20 weeks using a UVP XX-15MR Bench Lamp, 302 nm (Analytik Jena). The lamp was calibrated using a digital light meter (International Light Technologies. Mouse back skin was shaved with an electric razor before each UVB treatment. Mice were checked for skin cancer development 3 times a week and harvested 20 weeks after DMBA unless otherwise specified.

### Calcipotriol-plus–5-FU topical treatment in vivo.

For WT or IL24^KO^ mice on the C57BL/6 background, mice received topical calcipotriol (20 nmol) in ethanol plus 0.5 % 5-FU cream, ethanol-plus–5-FU, calcipotriol-plus–control cream, or control treatment 3 times a week starting in week 10 after DMBA until harvested week 20 after DMBA. SKH-1 mice received topical calcipotriol (50 nmol) in ethanol-plus–0.5% 5-FU cream or ethanol-plus–5-FU for 3 weeks from week 15 to week 18 after DMBA. WT mice on the FVB background received topical calcipotriol (40 nmol) in ethanol-plus–0.5% 5-FU, ethanol-plus–5-FU, calcipotriol-plus–control vehicle, or control treatment 3 times a week for 3 weeks (week 5–8 after DMBA).

### Adoptive T cell transfer.

For CD4^+^ T cell isolation, spleen and inguinal lymph nodes were digested with collagenase IV (Worthington Biochemical), and CD4^+^ T cells were negatively selected following the instruction of MojoSort Mouse CD4 T Cell Isolation Kit (BioLegend). CD3^+^CD4^+^CD45^+^ T cells were stained (Supplemental Table 3) and enriched with SH800 Cell Sorter (Sony Biotechnology). When donor mice were Tslp^tg^ and Tslpr^KO^ mice, they were irradiated with 450-cGy 2 days before adoptive T cell transfer. A day after adoptive T cell transfer, DMBA was applied. CD4^+^ T cells were injected intravenously. Tslp^tg^ Tslpr^KO^ mice received 2.0 × 10^6^ of adoptive CD4^+^ T cell transfer from Ly5.1 WT or Tslpr^KO^ mice. Tslp^tg^ Rag1^KO^ and Rag1^KO^ mice received 3.0 × 10^6^ of adoptive CD4^+^ T cell transfer from WT mice.

### Plasma TSLP ELISA.

Plasma samples were collected from the open-label trial participants before (day 0) and after treatment (day 7). Plasma TSLP levels were measured following the Human TSLP Quantikine ELISA kit instructions (R&D Systems). Samples from 4 participants who were treated for all 4 anatomical sites (face, scalp, RUE, and LUE) were included in the study.

### Study approval.

Human studies were reviewed and approved by the IRB of Washington University in St. Louis and Massachusetts General Hospital. Animal studies were approved by the IACUC of Massachusetts General Hospital.

### Statistics.

All bar graphs and dot plots show mean + SD. A 2-tailed Mann-Whitney *U* test was used as the significance test for nonpaired 2-group comparison. A 2-tailed paired *t* test was used for paired 2 group comparison. Kruskal-Wallis test with Dunn’s multiple comparison test was used for more than 2 group comparison. 2-way ANOVA with Dunnett’s multiple comparison test was used for tumor count over time analysis. GraphPad Prism 10 was used for statistical analysis. *P* < 0.05 was considered significant.

### Data availability.

The RNA sequencing data can be accessed from NCBI database, GEO accession no: GSE255479. Values for all data points found in graphs can be found in the supplemental Supporting Data Values file. Additional data related to this paper may be requested from the corresponding author.

## Author contributions

SD conceived the study. TO and SD designed the experiments. TO, SSS, HGS, TL, VSOG, MM, KET, LSI, DNC, and MA performed the experiments and analyzed the data. NSR, MT, LAC, and SD performed the clinical studies. TO and SD interpreted the data and wrote the manuscript. LE, SKD, and PBF contributed to Ad.IL-24 experiments, interpretation of IL-24 data, and writing of the manuscript.

## Supplementary Material

Supplemental data

Supporting data values

## Version 1 01/02/2025

Electronic publication
