# Supplementary material for: T helper 2 cell–directed immunotherapy eliminates precancerous skin lesions
Source: J Clin Invest. 2025 Jan 2;135(1):e183274. doi: 10.1172/JCI183274 (PMC11684800; doi:10.1172/JCI183274)
Supplement: Supplemental data [file jci-135-183274-s130.pdf]

Supplemental Material for

T helper 2 cell-directed immunotherapy eliminates pre-cancerous skin lesions

Tomonori Oka, Sabrina S. Smith, Heehwa G. Son, Truelian Lee, Valeria S. Oliver-Garcia, Mahsa Mortaja, Kathryn E. Trerice, Lily S. Isakoff, Danielle N. Conrad, Marjan Azin, Neel S. Raval, Mary Tabacchi, Luni Emdad, Swadesh K. Das, Paul B. Fisher, Lynn A. Cornelius, Shadmehr Demehri*

*Author for correspondence:
Shadmehr Demehri, M.D., Ph.D.
Department of Dermatology and MGH Cancer Center
Building 149 13th Street, 3rd floor
Charlestown MA 02129
Phone: 617-643-6436, Fax: 617-726-4453
Email: sdemehri1@mgh.harvard.edu

Supplemental Figures

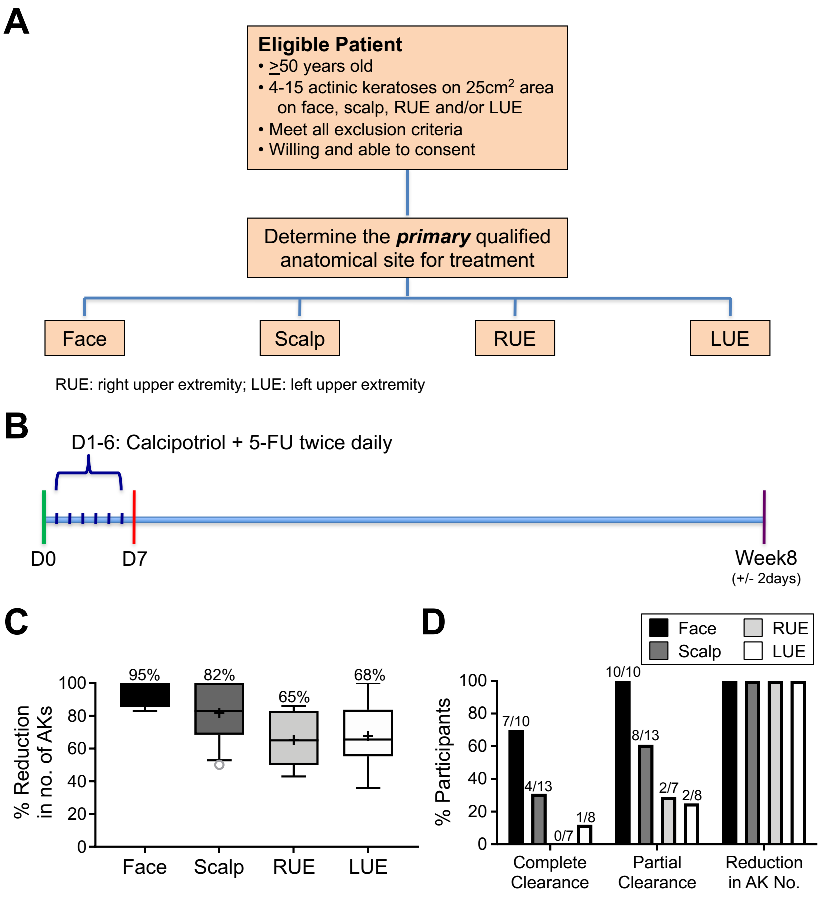

Supplemental Figure 1. Summary of calcipotriol plus 5-FU immunotherapy open-label trial design and clinical outcomes. (**A**) Schematic diagram of calcipotriol plus 5-FU open-label trial eligibility criteria and treated anatomical sites. RUE: right upper extremity, LUE: left upper extremity. (**B**) Schematic diagram of the timeline of the clinical visits and treatment. (**C**) % reduction in the number of AKs on the four anatomical sites treated with calcipotriol plus 5-FU at week 8 post-treatment. The average % reduction is listed on the graph. (**D**) % participants with complete (100%) and partial (> 75%) AK clearance on each of the four anatomical sites treated with calcipotriol plus 5-FU at week 8 post-treatment. The number of participants who showed complete and partial AK clearance per total participants for each anatomical site is listed on the graph.

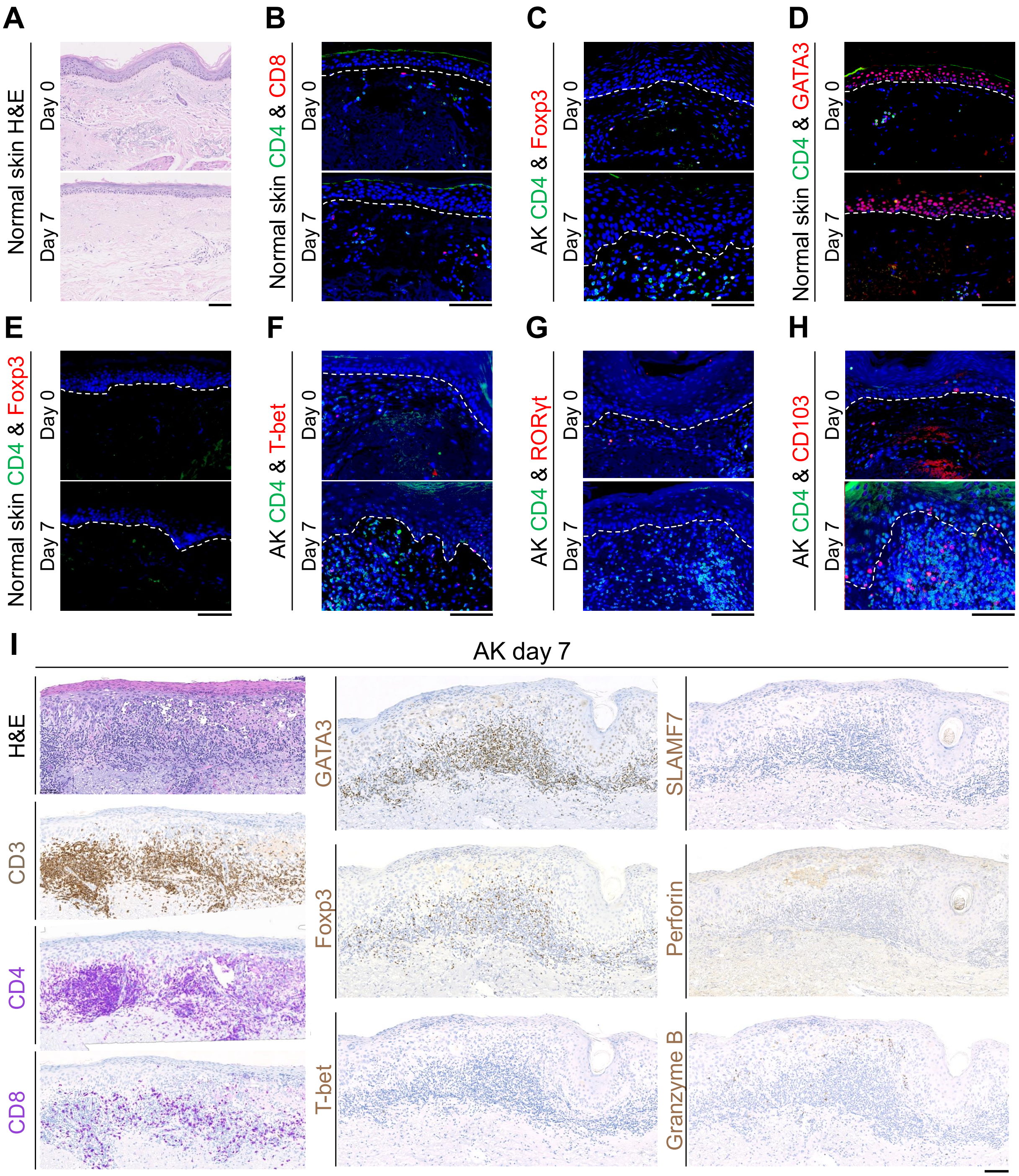

Supplemental Figure 2. Calcipotriol plus 5-FU immunotherapy activates Th2 immunity in AKs. (**A**) Representative H&E-stained normal skin before (day 0) and after (day 7) calcipotriol plus 5-FU treatment. (**B**) Representative images of CD4/CD8-stained normal skin before (day 0) and after (day 7) calcipotriol plus 5-FU treatment. Note that $CD4^+$ and $CD8^+$ cells are $CD3^+$ T cells.

(**C**) Representative images of CD4/Foxp3-stained AKs before (day 0) and after (day 7) calcipotriol plus 5-FU treatment. Note that $Foxp3^+CD4^+$ cells are $CD3^+$ T cells. (**D**) Representative images of CD4/GATA3 stained AKs before (day 0) and after (day 7) calcipotriol plus 5-FU treatment. Note that $GATA3^+CD4^+$ cells are $CD3^+$ T cells. (**E**) Representative images of CD4/Foxp3-stained normal skin before (day 0) and after (day 7) calcipotriol plus 5-FU treatment. Note that $Foxp3^+CD4^+$ cells are $CD3^+$ T cells. (**F**) Representative images of CD4/T-bet-stained AKs before (day 0) and after (day 7) calcipotriol plus 5-FU treatment. Note that T-bet^+CD4^+ cells are $CD3^+$ T cells. (**G**) Representative images of CD4/RORγt stained AKs before (day 0) and after (day 7) calcipotriol plus 5-FU treatment. Note that $ROR\gamma t^+CD4^+$ cells are $CD3^+$ T cells. (**H**) Representative images of CD4/CD103 stained AKs before (day 0) and after (day 7) calcipotriol plus 5-FU treatment. Note that $CD103^+CD4^+$ cells are $CD3^+$ T cells. (**I**) Representative images of H&E, CD3, CD4, CD8, GATA3, Foxp3, T-bet, signaling lymphocytic activation molecule family member 7 (SLAMF7), Perforin, and Granzyme B stained AKs after calcipotriol plus 5-FU treatment. Dashed lines mark the epidermal basement membrane in immunofluorescence images. Scale bars: 100 μm.

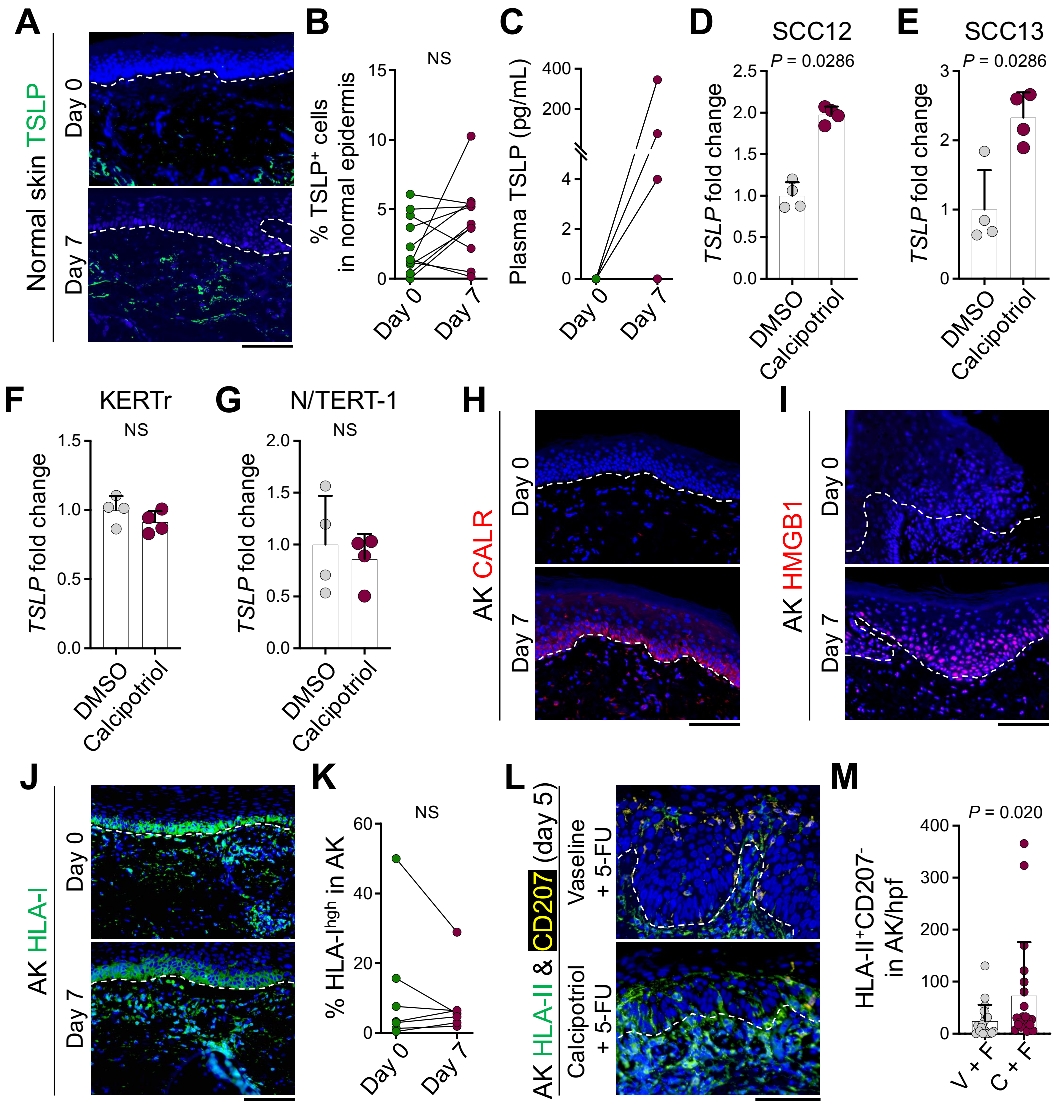

Supplemental Figure 3. Calcipotriol plus 5-FU treatment heightens AK immunogenicity. (**A**) Representative images of TSLP-stained normal skin before (day 0) and after (day 7) calcipotriol plus 5-FU treatment. (**B**) Quantification of TSLP$^+$ keratinocytes as percent DAPI$^+$ epidermal cells in normal skin before (day 0) and after (day 7) calcipotriol plus 5-FU treatment. Each dot represents a sample (n = 10 subjects at each timepoint, NS: not significant, paired t test). (**C**) Quantification of plasma TSLP levels before (day 0) and after (day 7) calcipotriol plus 5-FU treatment. Each dot represents a plasma sample (n = 4 subjects at each time point). (**D-G**) Quantification of *TSLP* mRNA expression in SCC12 (D), SCC13 (E), KERTr (F), and N/TERT-1

(G) cells treated with 1 μM calcipotriol for 24 hr. Each dot represents a biological replicate (*n* = 4 in each group, NS: not significant, Mann-Whitney *U* test). (**H-J**) Representative images of CALR (H), HMGB1 (I), and HLA-I (J) stained AKs before (day 0) and after (day 7) calcipotriol plus 5-FU treatment. (**K**) Quantification of HLA-I^{high} cells as percent DAPI^{+} keratinocytes in AKs. Each dot represents an AK sample (*n* = 8 subjects at each timepoint, NS: not significant, paired *t* test). (**L**) Representative images of HLA-II/CD207-stained AKs after Vaseline plus 5-FU versus calcipotriol plus 5-FU treatment. (**M**) Quantification of HLA-II^{+}CD207^{-} cells in AKs after Vaseline plus 5-FU (V + F) versus calcipotriol plus 5-FU (C + F) treatment. Each dot represents an AK sample (*n* = 20 in each group, Mann-Whitney *U* test). Bar graphs show mean + SD. Dashed lines mark the epidermal basement membrane in immunofluorescence images. Scale bars: 100 μm.

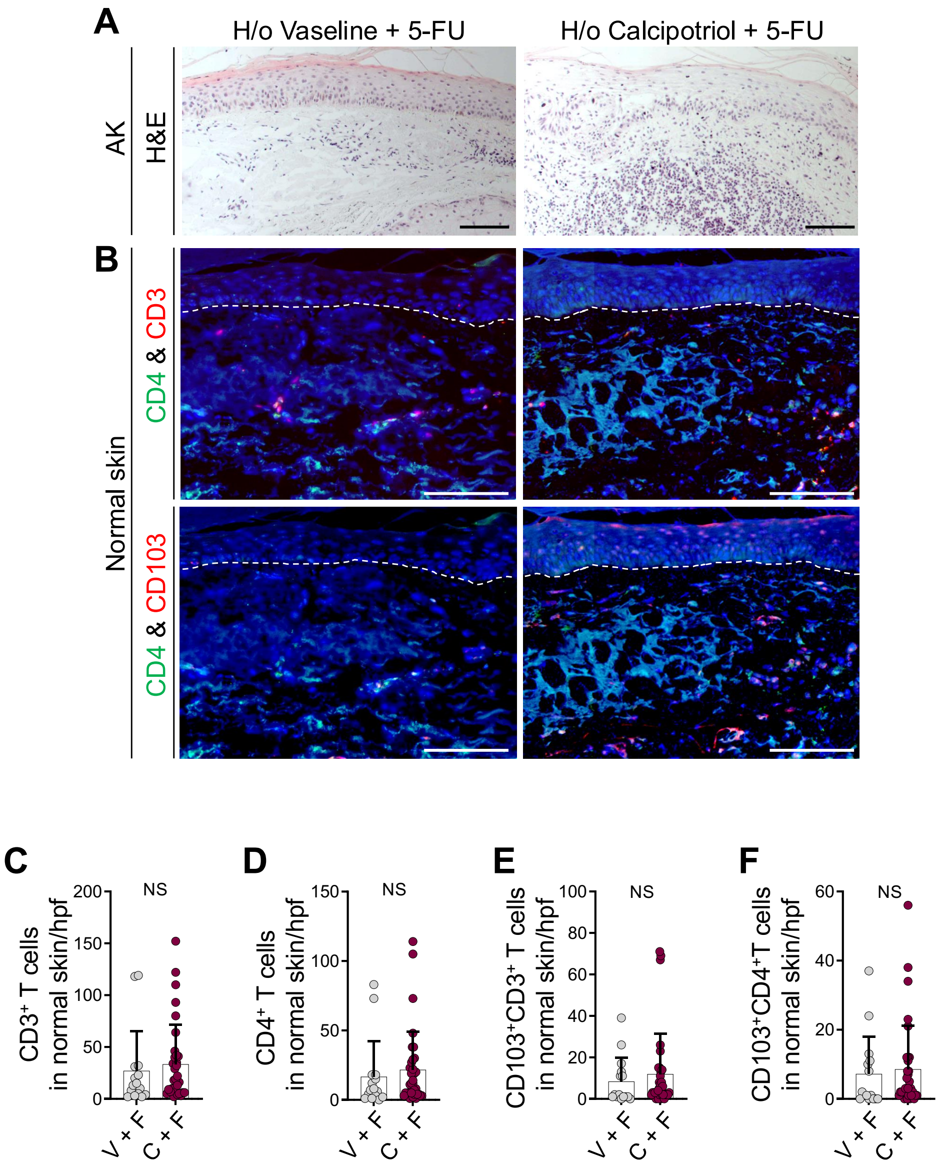

Supplemental Figure 4. T cell immunity induced by calcipotriol plus 5-FU treatment does not leave elevated T cell infiltrate in the normal skin over 5 years after treatment. (**A**) Representative image of H&E-stained AKs from participants with a history of (H/o) Vaseline plus 5-FU versus calcipotriol plus 5-FU treatment in the randomized clinical trial. (**B**) Representative images of CD4/CD3 and CD4/CD103 stained normal skin from participants with a history of treatment with Vaseline plus 5-FU versus calcipotriol plus 5-FU in the randomized clinical trial. (**C-F**) Quantification of $CD3^+$ T cells (C), $CD4^+$ T cells (D), $CD3^+CD103^+$ T cells (E), and

$CD103^+CD4^+$ T cells (F) in the normal skin from participants with a history of Vaseline plus 5-FU versus calcipotriol plus 5-FU treatment. Each dot represents cell counts from an hpf image. Three hpf images are included per sample (*n* = 5 in Vaseline plus 5-FU group, *n* = 11 in calcipotriol plus 5-FU group, NS: not significant, Mann-Whitney *U* test). Bar graphs show mean + SD. Dashed lines mark the epidermal basement membrane in immunofluorescence images. Scale bars: 100 µm.

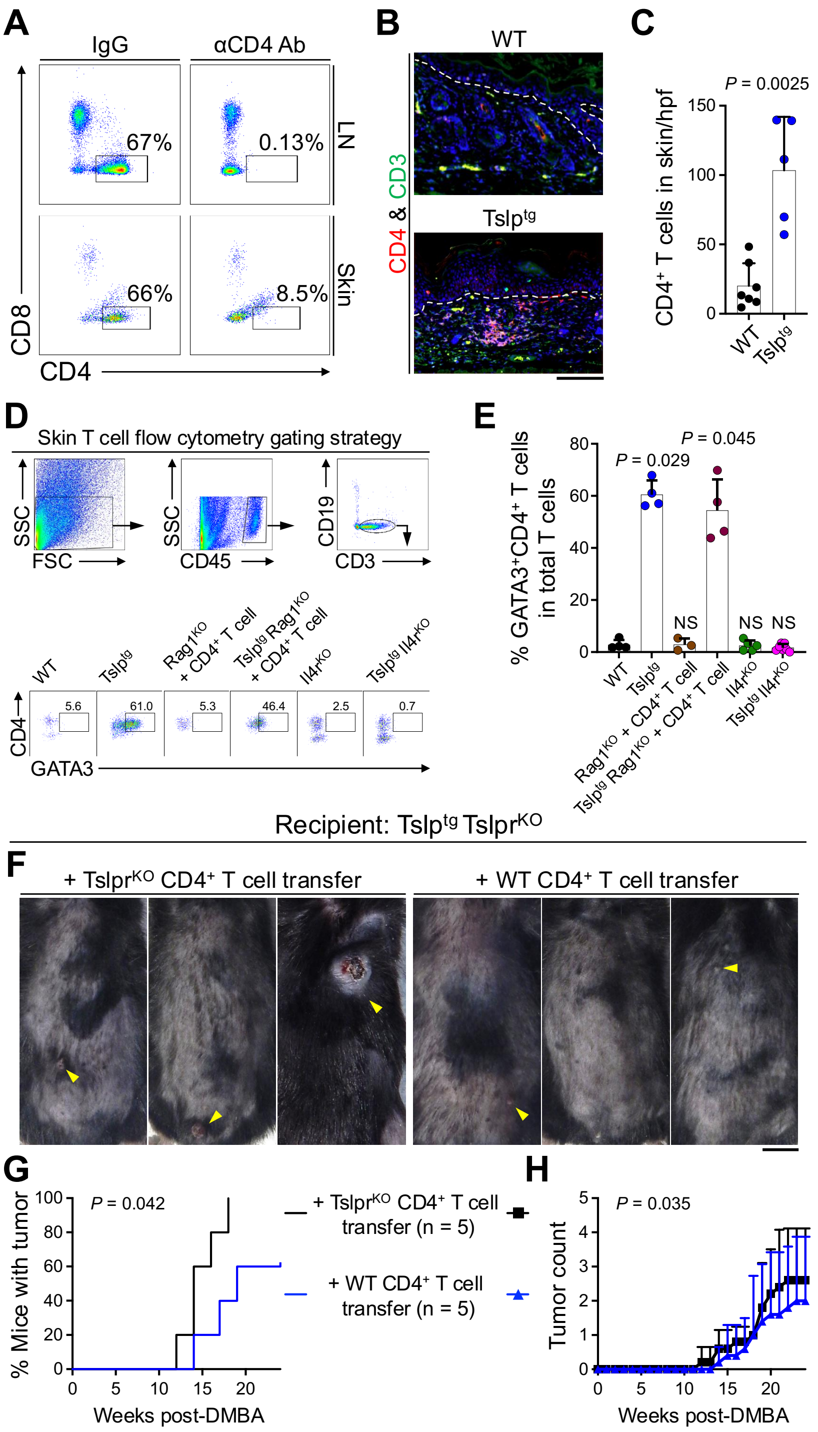

Supplemental Figure 5. TSLP induction in keratinocytes prevents skin cancer development in a Th2 cell-dependent manner. (**A**) Representative flow cytometry plots of CD4 and CD8 expression on CD45^{+}CD3^{+} T cells isolated from lymph nodes (LN) and skin after anti-CD4

antibody (αCD4 Ab) treatment. (**B**) Representative images of CD4/CD3-stained back skin of WT and Tslp^{tg} mice at week 19 post-DMBA (dashed lines mark the epidermal basement membrane, scale bar: 100 μm). (**C**) Quantification of CD4^{+} T cells in the back skin of WT and Tslp^{tg} mice. Each dot represents a mouse (WT: *n* = 7, Tslp^{tg}: *n* = 5, Mann-Whitney *U* test). (**D**) Representative flow cytometry plots demonstrating the gating strategy to identify CD3^{+} T cells in back skin and representative flow cytometry plots of GATA3 and CD4 expression in CD3^{+} T cells isolated from the back skin of mice at week 19 post-DMBA. (**E**) Quantification of GATA3^{+}CD4^{+} T cells per total T cells at week 19 post-DMBA in the back skin of mice with different genotypes. Each dot represents a mouse (WT: *n* = 4, Tslp^{tg}: *n* = 4, Rag1^{KO} + CD4: *n* = 3, Tslp^{tg} Rag1^{KO} + CD4: *n* = 4, Il4r^{KO}: *n* = 5, Tslp^{tg} Il4r^{KO}: *n* = 7, NS: not significant, Kruskal-Wallis test with Dunn's multiple comparison test). (**F**) Representative photographs of the back skin of Tslp^{tg} Tslpr^{KO} mice that received Tslpr^{KO} versus WT CD4^{+} T cell transfer. Yellow arrows point to skin tumors (scale bar: 1 cm). (**G, H**) Time to tumor onset (G, log-rank test) and the number of tumors per mouse over time (H, two-way ANOVA with Dunnett's multiple comparison test) in Tslp^{tg} Tslpr^{KO} mice that received Tslpr^{KO} versus WT CD4^{+} T cells. Bar graphs show mean + SD.

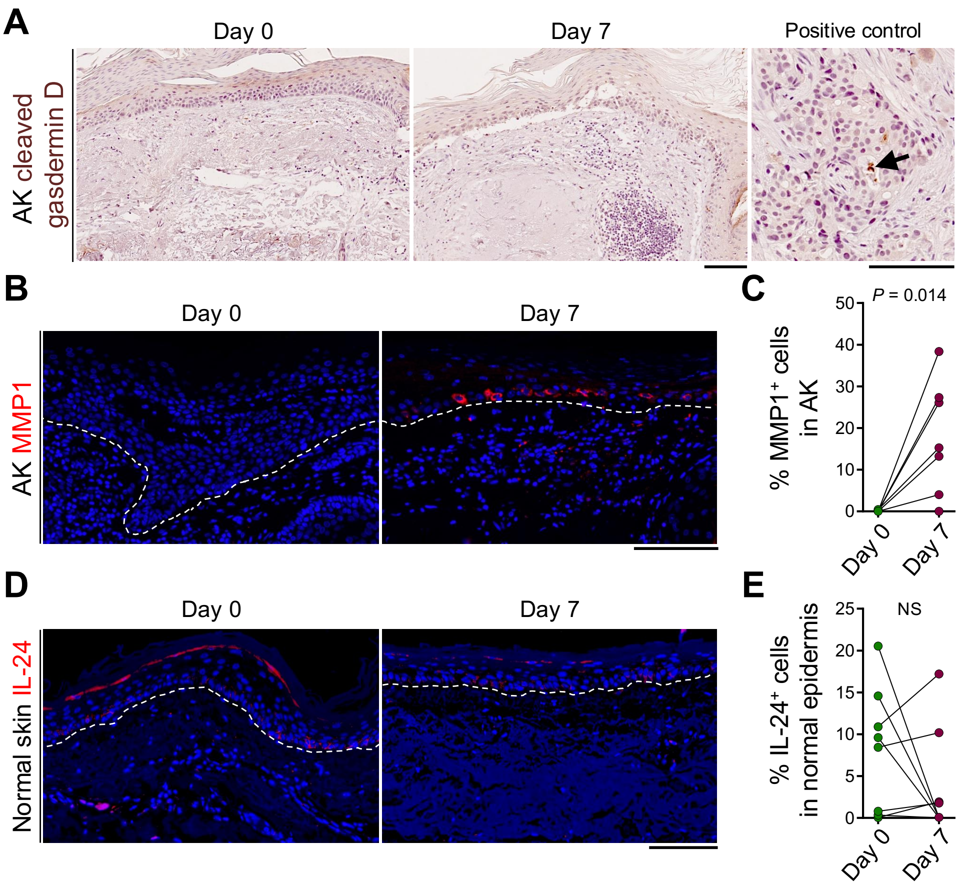

Supplemental Figure 6. Calcipotriol plus 5-FU treatment induces MMP1 expression in AK keratinocytes. (**A**) Representative images of cleaved gasdermin D-stained AKs before (day 0) and after (day 7) calcipotriol plus 5-FU treatment. The cleaved gasdermin D-stained breast cancer tissue section is shown as a positive control (the black arrow points to a cleaved gasdermin D^+ cell). (**B**) Representative images of MMP1-stained AKs before (day 0) and after (day 7) calcipotriol plus 5-FU treatment. (**C**) Quantification of $MMP1^+$ cells per total $DAPI^+$ cells in AK before (day 0) and after (day 7) calcipotriol plus 5-FU treatment. Each dot represents an AK sample (*n* = 8 subjects at each timepoint, paired *t* test). (**D**) Representative images of IL-24-stained normal skin before (day 0) and after (day 7) calcipotriol plus 5-FU treatment. (**E**) Quantification of IL-24^+ cells per total $DAPI^+$ epidermal cells in normal skin before (day 0) and after (day 7) calcipotriol plus 5-FU treatment. Each dot represents a normal skin sample (*n* = 8 subjects at each timepoint, NS:

not significant, paired *t* test). Dashed lines mark the epidermal basement membrane in immunofluorescence images. Scale bars: 100 μm.

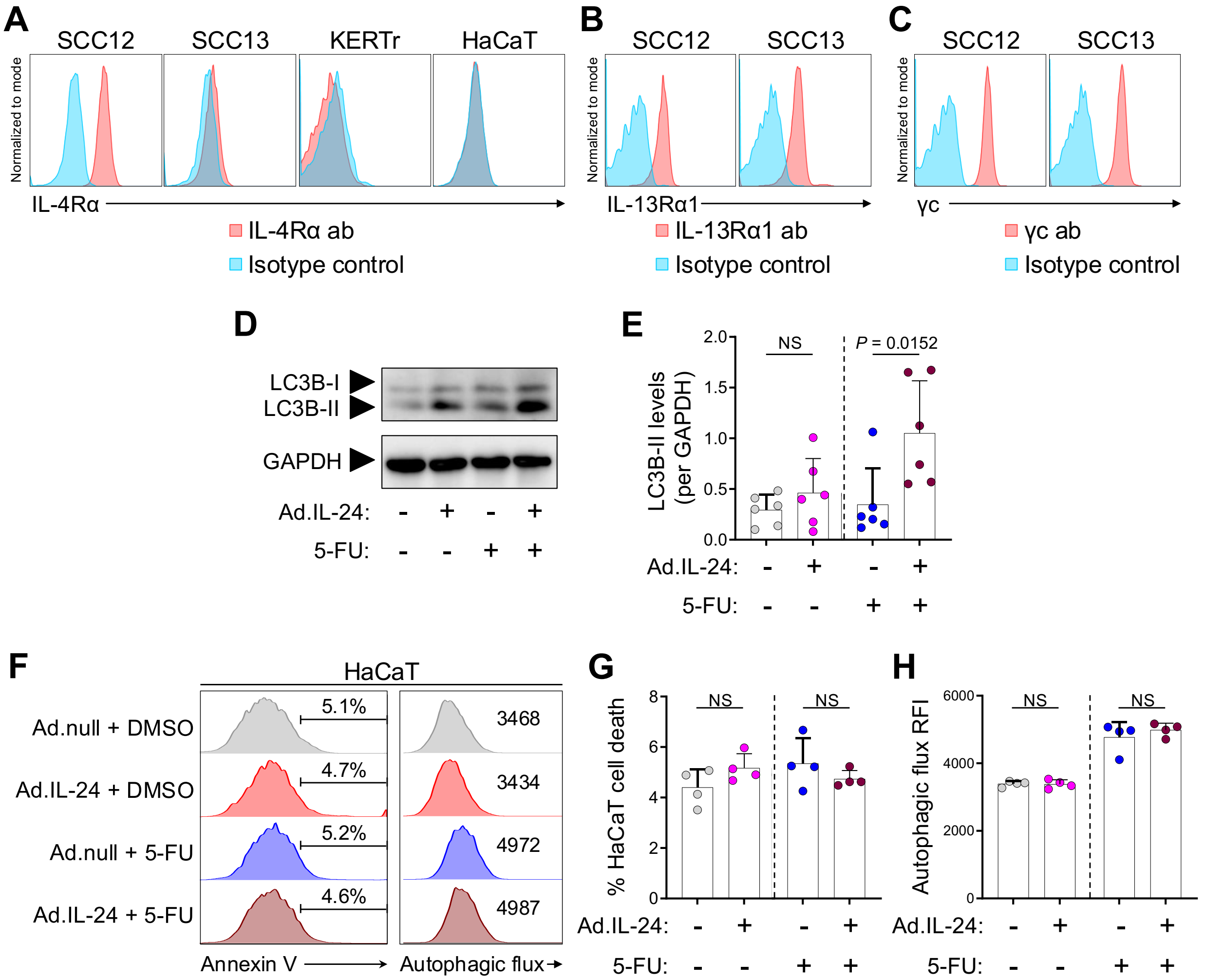

Supplemental Figure 7. SCC cells express IL-4 receptor, and IL-24 causes cell death in a cancer-selective manner. (**A**) Representative flow cytometry plots of IL-4 receptor α on the surface of SCC and normal keratinocyte cell lines. (**B, C**) Representative flow cytometry plots of IL-13 receptor α1 (B) and γc receptor (C) on the surface of SCC cell lines. (**D**, **E**) Representative immunoblot of LC3B-I, LC3B-II, and GAPDH (D), and quantification of LC3B-II protein levels (E) in SCC12 cells infected with adenovirus vector overexpressing IL-24 (Ad.IL-24) and treated with 5-FU compared with control vector (Ad.null) and DMSO. GAPDH is used as the control housekeeping protein (n = 6 in each group, NS: not significant, Mann-Whitney *U* test). (**F-H**) Representative flow cytometry histogram of annexin V and autophagic flux (F), and quantification of % cell death (G) and RFI of autophagic flux (H) on HaCaT cells infected with adenovirus type

5 vector expressing mda-7/IL-24 (Ad.IL-24) and treated with 5-FU compared with control vector (Ad.null) and DMSO (*n* = 4 in each group, NS: not significant, Mann-Whitney *U* test). Bar graphs show mean + SD.

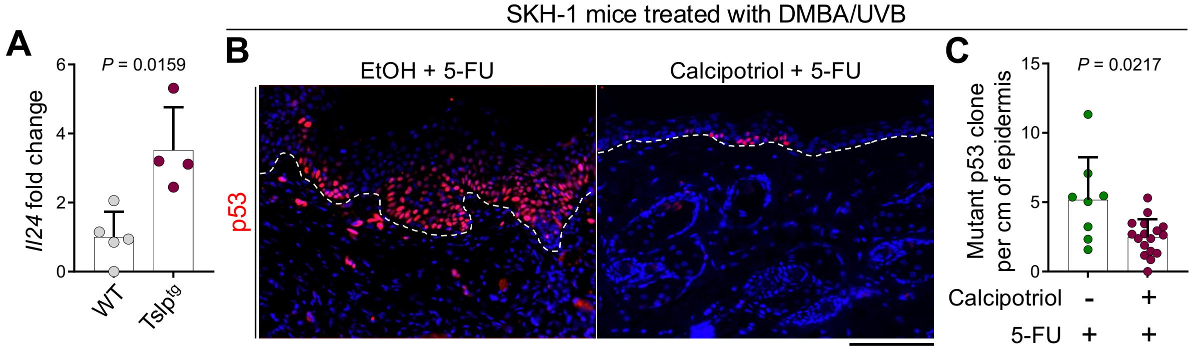

Supplemental Figure 8. Calcipotriol plus 5-FU immunotherapy prevents premalignant p53 mutant clones. (**A**) Quantification of *Il24* mRNA expression in the skin of WT and Tslp^{tg} mice. Each dot represents a mouse (WT: *n* = 5, Tslp^{tg}: *n* = 4, Mann-Whitney *U* test). (**B**) Representative images of p53-stained back skin of hairless immunocompetent SKH-1 mice treated with EtOH plus 5-FU versus calcipotriol plus 5-FU during DMBA/UVB skin carcinogenesis protocol at week 25 post-DMBA. Topical treatments were performed three times per week between week 15 and 18 post-DMBA. (**C**) Quantification of mutant p53 clones per cm of the epidermis in the back skin of SKH-1 mice treated with EtOH plus 5-FU or calcipotriol plus 5-FU at week 25 post-DMBA. Each dot represents a mouse (EtOH plus 5-FU: *n* = 8, calcipotriol plus 5-FU: *n* = 17, Mann-Whitney *U* test). Bar graphs show mean + SD. Dashed lines mark the epidermal basement membrane in immunofluorescence images. Scale bar: 100 μm.

Supplemental Tables

Supplemental Table 1. The demographic of the participants in the calcipotriol plus 5-FU immunotherapy open-label trial.

	Calcipotriol plus 5-FU (n = 18)
Gender, number of males, (% Males)	18 (100%)
Age, median, (range), year	68.5 (57-85)
Anatomical sites treated[#] n (%)	
Face	10 (56%)
Scalp	13 (72%)
RUE	7 (39%)
LUE	8 (44%)
Baseline AK count, median (interquartile range)	
Face	13 (10)
Scalp	23 (20)
RUE	21 (26)
LUE	14 (16)

[#]: A given participant may be counted in more than one treated anatomical site.

Supplemental Table 2. Genes upregulated in AKs after calcipotriol plus 5-FU treatment at day 5 compared with both before treatment (day 0) and after Vaseline plus 5-FU treatment at day 5. The fold change shown is calculated based on the comparison with before treatment (day 0).

Gene name	Fold change (log2)
MMP1	7.09
MMP3	6.96
FTHL17	4.53
FAM127A	4.44
HP	4.25
APOBEC3A	3.99
SMIM3	3.53
PI15	3.49
IL24	3.46
DEFB4B	3.46

Supplemental Table 3. Key resources list.

REAGENT or RESOURCE	SOURCE	IDENTIFIER
Histology antibodies (Human primary antibodies)		
CD3	Abcam	Cat#ab11089; RRID: AB_2889189
CD4	Thermo Fisher Scientific	Cat# MA1-39582; RRID: AB_10986805
CD8	Cell Signaling Technology	Cat#70306; RRID: AB_2799781
CD103	Abcam	Cat# ab129202; RRID: AB_11142856
CD207	Novus Biologicals	Cat# DDX0362P; RRID: AB_2892751
HLA-DP, DQ, DR (HLA-II)	Dako	Cat# M0775; RRID:AB_2313661
HLA Class 1 ABC	Abcam	Cat#ab70328; RRID: AB_1269092
GATA3	BioCare Medical	Cat#CM405A; RRID:AB_10895444
RORγt	BioCare Medical	Cat# 3208; RRID:AB_2924308
T-bet	Cell Marque	Cat# 368R-7; RRID:N/A
Foxp3	eBioscience	Cat#14-4776; RRID:AB_2865086
SLAMF7	BioLegend	Cat#331802; RRID:AB_961330
Perforin	R&D Systems	Cat# MAB8011; RRID:N/A
Granzyme B	Cell Signaling Technology	Cat# 46890; RRID:AB_2799313
TSLP	Abcam	Cat# ab47943; RRID:AB_883272
ANXA1	Cell Signaling Technology	Cat# 32934; RRID:AB_2799031
CALR	Cell Signaling Technology	Cat# 12238; RRID:AB_2688013
HMGB1	Cell Signaling Technology	Cat# 6893; RRID:AB_10827882
Cleaved caspase 7	Cell Signaling Technology	Cat# 8438; RRID:AB_11178377
Cleaved caspase 3	Cell Signaling Technology	Cat# 9664; RRID:AB_2070042
LC3B	Cell Signaling Technology	Cat# 3868; RRID:AB_2137707
IL-24	Abcam	Cat# ab115207; RRID:AB_10862303

Cleaved gasdermin D	Cell Signaling Technology	Cat# 36425; RRID:AB_2799099
MMP1	Abcam	Cat# ab137332; RRID:AB_2889296
Histology antibodies (Mouse primary antibodies)		
CD3	Abcam	Cat#ab11089; RRID: AB_2889189
CD4	Abcam	Cat#ab183685; RRID: AB_2686917
p53	Leica	Cat# P53-CM5P; RRID:AB_2744683
Histology antibodies (secondary antibodies)		
Goat anti Mouse IgG (H+L), Multi-Species SP ads-AF488	SouthernBiotech	Cat# 1038-30; RRID: AB_2794366
Goat anti Rabbit IgG (H+L) Antibody, Alexa Fluor 568	Abcam	Cat# ab175471; RRID:AB_2576207
Goat anti Rat IgG (H+L) Highly Cross-Adsorbed Secondary Antibody, Alexa Fluor Plus 647	Thermo Fisher Scientific	Cat# A48265; RRID:AB_2895299
Goat anti Rabbit IgG (H+L) Highly Cross-Adsorbed Secondary Antibody, Alexa Fluor 488	Thermo Fisher Scientific	Cat# A11034; RRID: AB_2576217
Flow cytometry antibodies		
Mouse CD45 (BV605)	BioLegend	Cat#103155; RRID:AB_2650656
Mouse CD3 (APC-Cy7)	BioLegend	Cat#100330; RRID:AB_1877171
Mouse CD19 (PE-Cy7)	Thermo Fisher Scientific	Cat#25-0193-82; RRID:AB_657663
Mouse CD4 (PerCP-Cy5.5)	BioLegend	Cat#100434; RRID:AB_893330
Mouse CD8 (BUV395)	BD Biosciences	Cat#740268; RRID:AB_2687927
Mouse GATA3 (BV421)	BioLegend	Cat#653814; RRID:AB_2563221
Human IL-4rα (CD124) (PE)	BioLegend	Cat#355003; RRID: AB_11219385
Human IL-13rα1 (CD213a1) (APC)	BioLegend	Cat#360405; RRID: AB_2562625
Human Common γ chain (CD132) (APC)	BioLegend	Cat#338607; RRID: AB_2123585
Bacterial and virus strains		
Ad.IL-24 (Ad.mda-7)	N/A	
Ad.null	N/A	
Chemicals, peptides, and recombinant proteins		
In vitro reagents		
Calcipotriol	Sigma-Aldrich	Cat#C4369
5-FU	Sigma-Aldrich	Cat#F6627
Recombinant human IL-4	BioLegend	Cat#574004

Recombinant human IL-13	BioLegend	Cat#571102
3-Methyladenine, autophagy inhibitor	Sigma-Aldrich	Cat#M9281
DMEM	Thermo Fisher Scientific	Cat#11-965-118
Keratinocyte SFM	Thermo Fisher Scientific	Cat#17005-042
In vivo reagents		
7,12-dimethylbenz(a)anthracene	Sigma-Aldrich	Cat#D3254-1G
Phorbol 12-myristate 13-acetate	Sigma-Aldrich	Cat#P8139-25MG
Anti-CD4 Rat Monoclonal Antibody	Bio X Cell	Cat#BE0003-1; RRID: AB_1107636
Rat immunoglobulin	Sigma-Aldrich	Cat#I4131-100MG
Calcipotriol	Sigma-Aldrich	Cat#C4369
Fluorouracil Topical Cream USP, 5%	Taro Pharmaceutical Industries, Ltd.	Cat# FLUOROURACIL CREAM USP
Histology reagents		
Paraformaldehyde	Sigma-Aldrich	Cat#P6148
Triton-X	Thermo Fisher Scientific	Cat#BP151
Citrate-Based Antigen Unmasking Solution	Vector Laboratories	Cat#H-3300-250
Tris-Based Antigen Unmasking Solution	Vector Laboratories	Cat#H-3301-250
Tween 20	Sigma-Aldrich	Cat#P1379
Normal goat serum	Sigma-Aldrich	Cat#G9023
Bovine serum albumin	Thermo Fisher Scientific	Cat#BP1600
DAPI	Invitrogen	Cat#D3571
Fluoroshield histology mounting medium	Sigma-Aldrich	F6182-20ML
Cytoseal XYL	Thermo Fisher Scientific	Cat#8312-4
Opal 4-Color IHC Kit	PerkinElmer	Cat#NEL820001KT
Hematoxylin	Sigma-Aldrich	Cat#GHS132-1L
Eosin	Leica Biosystems	Cat#380619
Formaldehyde	MillioporeSigma	Cat#F8775
Flow cytometry reagents		
Collagenase IV	Worthington Biochemical	Cat#LS004188
MojoSort Mouse CD4 T cell isolation Kit	BioLegend	Cat#480033
Annexin V Apoptosis Detection Kit with PI	BioLegend	Cat#640914
CYTO-ID Autophagy detection kit	Enzo Biochem	Cat#ENZ-KIT175-0200
Newborn calf serum	Thermo Fisher Scientific	Cat#26010074
Sodium azide	Sigma-Aldrich	Cat#S2002-100G
Intracellular staining Perm Wash Buffer	BioLegend	Cat#421002
Fixation Buffer	BioLegend	Cat#420801
Zombie NIR Fixable Viability Kit	BioLegend	Cat#423106
Propidium iodide	Sigma-Aldrich	Cat#P4864-10ml

Quantitative PCR reagents		
SuperScripts III Reverse Transcriptase	Thermo Fisher Scientific	Cat#18080085
dNTP mixture	Bio Basic	Cat#DD0056
RNasin Ribonuclease Inhibitor	Promega	Cat#PR-N2515
Random Primers	Life Technologies	Cat#48190011
iTaq Universal SYBR green supermix	Bio-Rad Laboratories	Cat#1725121
ELISA kit		
Human TSLP ELISA kit	R&D Systems	Cat#DTSLP0
Critical commercial assays		
Quick-DNA/RNA Microprep Plus Kit	Zymo Research	Cat#D7005
Quick-RNA Purification Kit, Miniprep	Zymo Research	Cat#R1055
Deposited data		
RNA Sequencing datasets	This paper	Available at NCBI accession #GSE255479
Experimental models: Cell lines		
KERTr	ATCC	Cat#CRL-2309
HaCaT	N/A	
SCC12	N/A	
SCC13	N/A	
UW-BCC1	N/A	
N/TERT-1	N/A	
Software and algorithms		
Zen Blue 3	Zeiss	https://www.zeiss.com/microscopy/en/products/software/zeiss-zen.html
NDP.View2	Hamamatsu Photonics	https://www.hamamatsu.com/jp/en/product/life-science-and-medical-systems/digital-slide-scanner/U12388-01.html
FlowJo 10	BD Bioscience	https://www.flowjo.co
Prism 10	GraphPad	https://www.graphpad.com/scientific-software/prism/
Biorender	Biorender	https://biorender.com/
Halo AI	Indica Labs	https://indicalab.com/halo-ai/
Equipment		
Zeiss Axio Observer Z1	Zeiss	N/A
Zeiss Axio Scan.Z1	Zeiss	N/A
LSRFortessa X-20	BD Bioscience	N/A
QuantStudio 3/5	Thermo Fisher Scientific	N/A

UVP XX-Series Bench Lamp 115V	ThermoFisher Scientific	UVP95004208
Hand-Held Light Meter	InternationalLight Technologies	ILT2400
C1000 Touch Thermal Cycler	Bio-Rad	N/A
NanoDrop spectrophotometer	NanoDrop Technologies	ND-1000

PCR primers for quantitative PCR			
Target gene	Forward or Reverse	Sequence	Source
Human GAPDH	Forward	AATCCCATCACCATCTTCCA	Integrated DNA Technologies
Human GAPDH	Reverse	TGGACTCCACGACGTACTCA	Integrated DNA Technologies
Human TSLP	Forward	CCCAGGCTATTCGGAAACTCAG	Integrated DNA Technologies
Human TSLP	Reverse	CGCCACAATCCTTGTAATTGTG	Integrated DNA Technologies
Human IL24	Forward	CAGGCGGTTTCTGCTATTCC	Integrated DNA Technologies
Human IL24	Reverse	CATCCAGGTCAGAAGAATGTCC	Integrated DNA Technologies
Mouse Gapdh	Forward	AATGTGTCCGTCGTGGATCTGA	Integrated DNA Technologies
Mouse Gapdh	Reverse	GATGCCTGCTTCACCACCTTCT	Integrated DNA Technologies
Mouse Tslp	Forward	CCAGGCTACCCTGAAACTGA	Integrated DNA Technologies
Mouse Tslp	Reverse	TCTGGAGATTGCATGAAGGA	Integrated DNA Technologies
Mouse Il24	Forward	CAATTCCATGCTTCCCATTAGTG	Integrated DNA Technologies
Mouse Il24	Reverse	ATTTCTGCATCCAGGTCAGGAG	Integrated DNA Technologies
PCR primers for Genotyping			
$Tslp^{tg}$	Forward	TGATGGTGTGAGCAGGAGAG	Integrated DNA Technologies
$Tslp^{tg}$	Reverse	TGTTTTGGACTTCTTGTGCCATTTCCTGAG	Integrated DNA Technologies
$Rag1^{KO}$	Forward	CCGCTTCCATTGCTCAGCGG	Integrated DNA Technologies
$Rag1^{KO}$	Reverse	CACGTTCTGTGAACCATGCTCTATC	Integrated DNA Technologies
$Rag1^{WT}$	Forward	CCAGTAGATACCATTGCGAAGAGG	Integrated DNA Technologies
$Rag1^{WT}$	Reverse	CACGTTCTGTGAACCATGCTCTATC	Integrated DNA Technologies
$Il4r^{KO}$	Forward	GGTTGCAGGGAACAGCCCAGAAAAGTGAAG	Integrated DNA Technologies
$Il4r^{KO}$	Reverse	CCAGACTGCCTTGGGAAAAG	Integrated DNA Technologies

Il4r^{WT}	Forward	GGTTGCAGGGAACAGCCCAGAAAAGTGAAG	Integrated DNA Technologies
Il4r^{WT}	Reverse	TCCTCTGTGGGCTCAGAGTGACCATGAGAA	Integrated DNA Technologies
Tslpr^{KO}	Forward	AGCGTTGGCTACCCGTGATATTGCTGAAGAG	Integrated DNA Technologies
Tslpr^{KO}	Reverse	TCATGAACGACCACTTCCTATGTTGGACACG	Integrated DNA Technologies
Tslpr^{WT}	Forward	TGAACCAGTCATGGTCTGGGATGATCTTGC	Integrated DNA Technologies
Tslpr^{WT}	Reverse	ATACCCCAAGATACGCCCATACCCTAAGAC	Integrated DNA Technologies
Il24^{KO}	Forward	GAGCCAGATCACAGGAAGTGAGG	Integrated DNA Technologies
Il24^{KO}	Reverse	ACTTGCTTTAAAAAACCTCCCACA	Integrated DNA Technologies
Il24^{WT}	Forward	AAGAAGCCCCTGTGTGGTGTAGC	Integrated DNA Technologies
Il24^{WT}	Reverse	GGAAATAAACTTTTCAGCCTTAAGAGC	Integrated DNA Technologies
